# Delivering interventions to reduce the global burden of stillbirths: improving service supply and community demand

**DOI:** 10.1186/1471-2393-9-S1-S7

**Published:** 2009-05-07

**Authors:** Zulfiqar A Bhutta, Gary L Darmstadt, Rachel A Haws, Mohammad Yawar Yakoob, Joy E Lawn

**Affiliations:** 1 Division of Maternal and Child Health, The Aga Khan University, Karachi, Pakistan; 2 Department of International Health, Bloomberg School of Public Health, Johns Hopkins University, Baltimore, Maryland, USA; 3 Saving Newborn Lives/Save the Children-US, Cape Town, South Africa; 4 International Perinatal Care Unit, Institute of Child Health, London, UK; 5 Health Systems Research Unit, Medical Research Council of South Africa, South Africa

## Abstract

**Background:**

Although a number of antenatal and intrapartum interventions have shown some evidence of impact on stillbirth incidence, much confusion surrounds ideal strategies for delivering these interventions within health systems, particularly in low-/middle-income countries where 98% of the world's stillbirths occur. Improving the uptake of quality antenatal and intrapartum care is critical for evidence-based interventions to generate an impact at the population level. This concluding paper of a series of papers reviewing the evidence for stillbirth interventions examines the evidence for community and health systems approaches to improve uptake and quality of antenatal and intrapartum care, and synthesises programme and policy recommendations for how best to deliver evidence-based interventions at community and facility levels, across the continuum of care, to reduce stillbirths.

**Methods:**

We systematically searched PubMed and the Cochrane Library for abstracts pertaining to community-based and health-systems strategies to increase uptake and quality of antenatal and intrapartum care services. We also sought abstracts which reported impact on stillbirths or perinatal mortality. Searches used multiple combinations of broad and specific search terms and prioritised rigorous randomised controlled trials and meta-analyses where available. Wherever eligible randomised controlled trials were identified after a Cochrane review had been published, we conducted new meta-analyses based on the original Cochrane criteria.

**Results:**

In low-resource settings, cost, distance and the time needed to access care are major barriers for effective uptake of antenatal and particularly intrapartum services. A number of innovative strategies to surmount cost, distance, and time barriers to accessing care were identified and evaluated; of these, community financial incentives, loan/insurance schemes, and maternity waiting homes seem promising, but few studies have reported or evaluated the impact of the wide-scale implementation of these strategies on stillbirth rates. Strategies to improve quality of care by upgrading the skills of community cadres have shown demonstrable impact on perinatal mortality, particularly in conjunction with health systems strengthening and facilitation of referrals. Neonatal resuscitation training for physicians and other health workers shows potential to prevent many neonatal deaths currently misclassified as stillbirths. Perinatal audit systems, which aim to improve quality of care by identifying deficiencies in care, are a quality improvement measure that shows some evidence of benefit for changes in clinical practice that prevent stillbirths, and are strongly recommended wherever practical, whether as hospital case review or as confidential enquiry at district or national level.

**Conclusion:**

Delivering interventions to reduce the global burden of stillbirths requires action at all levels of the health system. Packages of interventions should be tailored to local conditions, including local levels and causes of stillbirth, accessibility of care and health system resources and provider skill. Antenatal care can potentially serve as a platform to deliver interventions to improve maternal nutrition, promote behaviour change to reduce harmful exposures and risk of infections, screen for and treat risk factors, and encourage skilled attendance at birth. Following the example of high-income countries, improving intrapartum monitoring for fetal distress and access to Caesarean section in low-/middle-income countries appears to be key to reducing intrapartum stillbirth. In remote or low-resource settings, families and communities can be galvanised to demand and seek quality care through financial incentives and health promotion efforts of local cadres of health workers, though these interventions often require simultaneous health systems strengthening. Perinatal audit can aid in the development of better standards of care, improving quality in health systems. Effective strategies to prevent stillbirth are known; gaps remain in the data, the evidence and perhaps most significantly, the political will to implement these strategies at scale.

## Introduction

**Figure 1:**
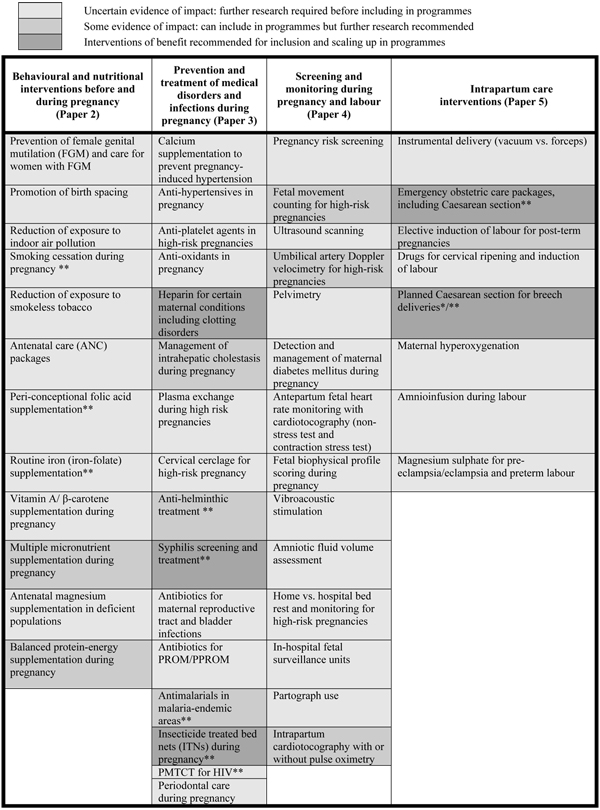
**Summary of evidence for all interventions to prevent stillbirth reviewed in this series (Papers 1–5)**. * Recommended only where access to referral-level care is good. ** Clear benefit for maternal and/or neonatal health.

The previous five papers in this series have focused on the global burden of stillbirths [1] and the evidence base for interventions [2-5] to reduce this burden. In order to prevent stillbirths, high-impact interventions must be effectively delivered through health systems and reach high coverage. Despite calls for action to improve stillbirth outcomes, the strategies for delivering such interventions in health systems and in communities remain unclear. Consensus is needed on priority interventions (Figure 1), but also on strategies to deliver them. In this paper, we focus on the evidence for key strategies for delivering effective interventions.

The interventions which need to be delivered in health systems have been reviewed in preceding papers in this series. Briefly, these interventions have included general supportive measures to improve environmental and social conditions as well as interventions that address maternal nutrition and prevention and management of maternal and fetal infections and conditions during pregnancy and childbirth. Based on our analysis of the evidence for impact of 60 different peri-conceptional, antenatal, and intrapartum maternal interventions on prevention of stillbirths, we categorised interventions into one of four levels according to the strength and quality of the evidence, and, therefore, the level of confidence in recommending interventions for wide-scale delivery in programmes (Figure 1). Five interventions of proven benefit in reducing stillbirths were identified: syphilis screening and treatment, use of insecticide-treated bednets in malaria-endemic areas during pregnancy, administration of heparin for certain maternal conditions including auto-immune and clotting disorders, and emergency obstetric care, including planned Caesarean section for breech delivery in settings where access to referral-level care is good. We presented caveats pertaining to the implementation of several of these interventions. Another 9 interventions were identified for which there was some evidence of impact, and we recommend that consideration be given to also including these in programmes; however, further research and monitoring of impact is required in programmatic settings.

Strategies to deliver those interventions with demonstrated or promising impact on stillbirths will need to employ a mix of service delivery modes, including family-community, outreach and clinical/facility-based platforms as used previously in the *The Lancet *Neonatal series and the World Bank Development report [6,7].

*Family-community interventions *can be taught or administered to mothers and/or other family members, and include the adoption of improved preventive practices and appropriate care-seeking for illness. Family and community care interventions include strategies for community mobilization and the empowerment of individuals and communities to demand quality health care services and find solutions to financial, logistical, and social barriers to care-seeking for maternal and neonatal illness. These services can be provided by various cadres of workers, and should be tailored to the community's social and cultural environment [8].

*Outreach interventions *may be provided through static health facilities or periodic community visits, and usually involve provision of a standardised intervention. These activities typically require less skill and training than clinical care services [8]. Community-based, outreach interventions and interventions delivered at primary health clinics or peripheral facilities and district hospitals can be packaged as primary care interventions [7,9].

*Facility-dependent clinical interventions *may be delivered at secondary or tertiary care settings. Secondary care is hospital-based and specialist-dependent, involving surgical or other advanced interventions that primary care providers cannot perform. Few of the interventions we considered require tertiary care, the most complex level of intervention demanding highly specialised, technology-dependent solutions [10].

The specific mix of interventions recommended for delivery at various levels of care will depend upon the level of and distribution of capabilities and resources within the local health system as well as local prevalence of risk factors for stillbirth. Some interventions, such as Caesarean section and amniotic fluid volume assessment, require resources based at well-equipped facilities, whereas interventions such as multiple micronutrient supplementation can be delivered via relatively straightforward outreach services.

In this paper, we examine a variety of intervention delivery strategies for prevention of stillbirth, including demand creation at the community level to improve access to and uptake of services, appropriate training of providers to perform these interventions, innovative ways of organising the health system and the ways in which care is delivered, and evaluative measures to improve quality of service.

## Methods

Methods utilised in the review portion of this paper follow the methods that have been described in detail in Paper 1 of this series [1]. We considered the rigour of studies we included, assigning a level of evidence (LOE) to each study we reviewed as well as a grade for the total body of evidence for a given intervention using the SIGN system. In this particular paper, we reviewed twelve cross-cutting interventions (see Table 1) that aim to improve access to and uptake of interventions, as well as quality of services delivered.

**Table 1:** Community and health-systems interventions to impact stillbirths reviewed in this paper

***Community demand creation strategies***
Emergency loan and insurance funds for emergency obstetric care
Financial incentives for care-seeking

***Human resource development and training***
Training of traditional birth attendants (TBAs) in clean delivery and referral
Training of other cadres of community health workers
Training of nurse-aides (including task-shifting) as birth attendants
Training to improve skills of professional midwives in antenatal and intrapartum care
Obstetric drills
Training in neonatal resuscitation for physicians and other health workers

***Health system organizational strategies***
Public-private partnerships to provide emergency obstetric care
Maternity waiting homes
Home birth with skilled attendance versus hospital birth for low-risk pregnancy

***Evaluation strategies***
Perinatal audit

Most of the interventions reviewed in this paper consider the impact of strategies of operationalising delivery of interventions, not the impact of biomedical or behavioural interventions themselves. In a few instances, studies we identified did not report impact on stillbirth or perinatal mortality specifically, yet contributed important information about innovative intervention delivery strategies that could be used to improve uptake or quality of interventions to prevent stillbirths. While we included these studies in our evaluation, only those interventions for which studies reported impact on perinatal mortality were included in summary tables; studies that did not report stillbirths or perinatal mortality outcomes were discussed wherever relevant in the text.

## Results

### Community demand creation strategies

In many settings, coverage of effective interventions that prevent stillbirth is low, in part because demand for these services is poor among the groups with the least resources, who stand to benefit most from accessing interventions [11]. Financial, geographic, and cultural barriers to care-seeking, as well as perceptions of poor quality of services at health facilities, discourage the use of services. Community demand creation refers not only to efforts to mobilise community awareness of health risks and promotion of best practices, but also to promote fiscal mechanisms to support uptake of these services [12]. Demand creation is most effective alongside supply-side efforts to strengthen health systems and improve quality of service provision in facilities.

#### Emergency loan/insurance funds for emergency obstetric care

##### Background

One of the major barriers to maternal and newborn health care is limited financial resources. A number of strategies have been developed to address demand-side barriers to accessing care, and thus incentivise care-seeking, especially in health emergencies. Strategies that have been employed in low-resource, rural settings include community emergency loan and insurance schemes. These schemes pool and manage capital to pay user/patient fees, transport and medication costs and follow-up care, as well as opportunity costs incurred during care-seeking such as missed wages, the combined costs of which can be catastrophic for families. Loans generally need to be repaid, whereas insurance schemes charge a fixed prepayment in exchange for the promise that a fraction or all of the cost of services will be reimbursed if utilised. These strategies spare families from the potentially catastrophic financial impact of obstetric complications [13], which have been documented to be as high as 34% of annual household income in Benin [14], and which are often higher at comprehensive essential obstetric care facilities that can provide emergency interventions such as Caesarean section and blood transfusion compared to basic obstetric care facilities (9 times higher in a recent study from Bangladesh) [15]. These schemes can incentivise care-seeking, particularly in emergencies.

##### Literature-based evidence

We evaluated the available evidence regarding the impact of community-based emergency loan or insurance schemes on maternal health and birth outcomes. Seven observational studies met our criteria for selection.

The studies we identified all described the implementation of community loan and insurance schemes for obstetric complications, but none reported the impact on stillbirths or perinatal mortality. Chiwuzie et al. [16] described a scheme to mobilise clans in Ekpoma, Nigeria to create emergency loan funds for obstetric complications, which occurred alongside upgrades to emergency obstetric services in local health facilities. Twelve of thirteen clans successfully launched loan funds and collected donations totaling US $793, of which 80% was contributed by community members. In the first year, 456 loans were requested, 83% were granted, and 93% were repaid in full. Loans were used to pay for emergency transport, medications, blood transfusions and hospital fees ***[LOE: 2+]***.

Two other studies reported the implementation of community loan schemes in Sierra Leone and Nigeria. The project in Nigeria [17] set up an emergency transport system that used private drivers charging a set fee for emergency transport and created a loan fund of US $20,500, from which 18 loans were made in 9 months ***[LOE: 2-]***. Data on repayment or outcomes were not published. In Sierra Leone, women from two chiefdoms that established community loan funds [18] increased their utilization of obstetric services at the local government hospital compared to utilization prior to the fund (5 women in 1992 versus 12 in 1993) ***[LOE: 2-]***.

Several community-based insurance schemes relying on voluntary, flat-rate annual contributions for membership have reported increases in skilled attendance at delivery [19-21], with one study in the Democratic Republic of Congo reporting rates of skilled attendance 7 times higher among members of the insurance scheme compared to non-members. A much larger, nationwide social insurance scheme to provide for maternal and child health care in Bolivia increased ANC coverage and skilled attendance at birth by 50% in public facilities [22].

##### Conclusion

None of the studies evaluated loans or insurance for emergency obstetric care and pregnancy outcomes, and many schemes at community level were too small to measure meaningful changes in mortality rates, but the potential of these interventions to improve utilization of facility-based services and thus reduce perinatal mortality seems promising. Available evidence indicates that with relatively little outside financial input, communities can successfully set up and administer loan funds for emergency obstetric transport and care, with relatively low default rates. Sustained long-term loan and insurance schemes will require continuing community involvement with strong leadership to raise and manage funds, follow-up on defaults, and maintain records [23]. These initiatives require sufficient resources to cover administrative costs to collect funds or insurance premiums and oversee their proper distribution. In addition, schemes may fail, especially if collection of funds is insufficient to cover costs or default rates are high, or may exclude the poorest individuals. In some of these areas, an alternative to these community-based schemes could potentially take the form of national or district-level government sponsorship and/or management of obstetric risk insurance programmes, a strategy which has been successful in increasing access to comprehensive essential obstetric care and postnatal care in Mauritania [24], and which waives costs of coverage for the poorest individuals. Further studies to assess the impact of loan and insurance schemes on maternal/neonatal health care access and stillbirth/perinatal outcomes are needed, as is operational research to identify best practices to administer these schemes.

#### Financial incentives for care-seeking

##### Background

In addition to community loan schemes, a number of other strategies have been developed to minimise financial barriers to care-seeking, protect families from catastrophic costs of obstetric emergencies, and stimulate demand among poor or otherwise marginalised women. These strategies include conditional cash transfers and voucher schemes. Conditional cash transfers provide money to individuals or families, on the condition of their using specific health services such as antenatal care (ANC), skilled birth attendance at a facility, or postnatal services [25]. Conditional cash transfers alone do not solve all access issues, as they are made after care is received and require that recipients cover transport expenses before receiving funds. However, conditional cash transfers can reduce long-term indebtedness because they can be used to repay emergency loans from family, neighbors, banks, or community schemes. Voucher schemes are another relatively new strategy to generate demand; women given vouchers at the community level can redeem them for pre-specified services at participating (contracted) health facilities [26]. These are particularly useful in non-cash economies, and limit expenditures to transport and opportunity costs.

##### Literature-based evidence

Nine studies were identified with relevance to utilization of antenatal and obstetric services and are discussed in this section; none reported impact on perinatal mortality outcomes.

In a review of conditional cash transfers to families in low-/middle-income countries, Lagarde et al. (2007) [27] assessed associated improvements in the health and education of children beneficiaries of the program. Although limited, the evidence suggested that these led to improved uptake of interventions and some health benefits. One study in this review of capped transfers in Brazil [28], in which mothers received funds after accessing care calculated based on whether they were pregnant, lactating, and/or the number of children they had under age 7, was associated with a 19-percentage-point increase in ANC attendance. Other observational studies of conditional cash transfers have shown increases in uptake of ANC. In Mexico, the Progresa/Oportunidades project documented increases in ANC utilization of 8 percent during the first trimester [29,30]; in Honduras, conditional cash transfers led to ANC coverage increases of 15–20 percent [31].

A national-level fiscal incentive programme introduced in 2005 by the Indian government under the umbrella of the National Rural Health Mission to promote facility-based deliveries, called the Janani Suraksha Yojana (JSY) Programme, provides cash assistance to poor rural pregnant women at childbirth and postnatally for their first and second pregnancy, with additional funds for emergency transport and Caesarean section [32]. Utilization of skilled care and facility-based services increased from 10.85 million beneficiaries in 2005–6 to 13.59 million in 2007–8 [33]. A similar program is in operation in Nepal, in which the government finances facility-based delivery in poor areas and provides conditional cash transfers to women who receive services at facilities as well as to their care providers [34].

Several ongoing projects are evaluating the ability of voucher systems to increase access to obstetric care, as well as to preventive interventions. In India, the Government of Gujarat introduced voucher schemes to increase the access of poor women to antenatal, obstetric and neonatal health care [35]. As part of a World Bank programme to improve pregnancy outcomes in western Uganda, 170,000 safe delivery vouchers were distributed to pregnant women covering services including Caesarean section at a number of public and private service providers [36]; no outcome data are yet available. In Tanzania, a nationwide voucher scheme was introduced to provide free or discounted insecticide-treated bed nets to pregnant women and mothers of young children to prevent malaria; although distribution of bed nets has been highly successful in preventing malaria and cost-effective, no data have yet been published evaluating the impact of the program on maternal or perinatal mortality [37,38]***[LOE: 2-]***.

##### Conclusion

The financial incentive schemes detailed here offer an opportunity to effectively target specific groups of individuals in a society, reduce reliance on cash in subsistence economies [25], and effectively promote uptake of services. To date, many of the studies testing various financial incentives to generate community demand for services have not published outcomes, barring quantification of the effect of these programs on birth outcomes. Most of the projects profiled have been funded by private or international donors or public-private partnerships rather than governments alone, and their effectiveness, cost, scalability, and sustainability are still unknown.

The incentive programmes detailed above do not cover time and transport costs, which can vary greatly among settings; for example, studies have indicated that combined time and transport costs range from 9–14% of total annual household expenditure in Nepal, compared to 65–93% in Tanzania [39,40]. Unless financial incentive programmes are expanded to include funds for emergency transport or are effectively integrated with sustainable community loan or insurance schemes, these costs are likely to continue to impede access. Voucher schemes run the risk of "leakage" (sale on the black market or use by non-targeted individuals) [38]. In health systems with user fees, community demand may be sufficient, and the population in need so broad, that merely rescinding such fees could improve rates of uptake of services and subsequent perinatal health outcomes [41]. Global interest in financial incentive strategies is burgeoning; programme managers should be encouraged to measure perinatal mortality outcomes, particularly stillbirths, wherever feasible.

### Human resource development and training

The critical role of formally trained professional health personnel – primarily physicians and nurses – in primary care and community settings is well recognised. Shortages of formally trained health workers in some countries has been underscored as a major barrier to implementation of key maternal and newborn interventions [42,43]. Delivering key interventions effectively requires proper training for these health workers as a means of promoting appropriate care, as well as providing adequate supervision and linkages with the formal health system. However, there are also numerous other cadres of health workers, including Traditional birth attendants (TBAs), midwives, other CHWs, and nurse aides, who are already active, or could potentially be utilised, in delivering interventions [44]. In this section, the potential roles and impact of these providers are examined.

#### Training TBAs in clean delivery and referral

##### Background

TBAs have a role in supporting women during labour but are generally not trained to deal with complications. TBAs are generally categorized as trained or untrained. Even so-called "trained TBAs" have often had a month or less of training and therefore cannot be defined as skilled attendants who should possess a minimum of skills, confidence and connectedness to the health system for management of complications. TBAs have often learned to assist births by apprenticeship to more experienced TBAs, often observing local traditions and customs, and may provide other postnatal services to women including caregiving and domestic chores. TBAs practice widely in many areas with poor access to facility-based care, and may be the birth attendant of choice even for women with access to facility-based care. Thus, the World Health Organization (WHO) had until recently promoted TBA training in clean delivery and referral of women with pregnancy and labour complications as a strategy to reduce maternal and neonatal mortality [45]. TBA training involves a short course of a few days to several months of biomedical training in clean delivery, cord care, and prevention of postpartum hemorrhage. Training may also include efforts to improve linkages between TBAs with the formal health care system through prevention and referral. However, while there is some evidence that TBA training can improve neonatal outcomes, there is no evidence that training reduces maternal mortality, and a dearth of evidence for impact on stillbirth outcomes.

##### Literature-based evidence

The review of literature identified 2 systematic reviews, as well as 8 other intervention and observational studies (Table 2). Sibley and Sipe [46] conducted a meta-analysis of the impact of training TBAs on a range of birth outcomes (17 studies), reporting a 6% decrease in perinatal mortality and an 11% decrease in birth asphyxia-associated mortality among mothers cared for or living in areas served by trained vs. untrained TBAs ***[LOE: 1+]***. An update of this analysis by the same authors was published as a Cochrane review [45] (4 trials, N = 2000 TBAs, N~27,000 women) (Additional file 1). The review included a trial by Jokhio et al [47] that reported significantly reduced stillbirth rates at births attended by trained versus untrained TBAs (adjusted OR = 0.69, 95% CI: 0.57–0.83, P < 0.001), perinatal mortality (adjusted OR = 0.70, 95% CI: 0.59–0.83, P < 0.001) and neonatal mortality (adjusted OR = 0.71, 95% CI: 0.61–0.82, P < 0.001). Another before-after intervention study in the same review [48], however, reported a non-significant impact on perinatal mortality of additional training of TBAs compared to basic training (24/203 vs. 37/318; OR = 1.02; 95% CI: 0.59–1.76).

**Table 2:** Impact of training traditional birth attendants on stillbirths and perinatal mortality

**Source**	**Location and Type of Study**	**Intervention**	**Stillbirths/Perinatal Outcomes**
***Reviews and meta-analyses***			

Sibley et al. 2007 [45]	Pakistan and rural Guatemala. Meta-analysis (Cochrane). 2 RCTs included (N = 18,699 pregnant women).	Assessed the effects of trained (intervention) vs. untrained (controls) traditional birth attendants.	SBR: adj. OR = 0.69 (95% CI: 0.57–0.83).

Sibley and Sipe 2004* [46]	24 countries and three regions. Meta-analysis (non-Cochrane). 60 studies included	Assessed the effects of the training of TBAs (intervention) vs. untrained TBA baseline (controls).	PMR: 8% reduction with TBA training (statistically significant) Birth asphyxia-associated PMR: 11% with TBA training (statistically significant).

***Intervention studies***			

Alisjahbana 1995 [54]	Indonesia (West Java). 2 districts. Longitudinal, intervention study. N = 3275 pregnant women (N = 2275 in the intervention district, and N = 1000 in the controls).	Assessed the impact of the intervention in a district where training was given at all levels of the health care system (informal and formal) and birthing homes were established in villages with special attention to referral, transportation, communication and appropriate case management. There was no intervention in the control district.	PMR: 99/2275 (43.5/1000) vs. 37/1000 (37/1000) in study and control villages respectively **[NS]**. There was a decrease in PMR in the intervention village with time, compared to no change in the control village.

Greenwood et al. 1990 [53]	Gambia (Farafenni area). 41 rural villages. Before-after intervention study.	Assessed the impact of primary heatlh care (PHC) programme in villages (intervention) vs. villages without the PHC programme (controls). Survey was also done for one year before and three years after the start of the programme.	SBR: 50/1000 (61/1220) vs. 51.9/1000 (37/712) in the PHC village vs. non-PHC villages in the post intervention period. (SBR increased in both PHC and non-PHC villages during first post-intervention year, possibly due to improved surveillance). PMR: 81.1/1000(99/1220) vs. 88.4/1000 (63/712) in PHC villages vs. non-PHC villages in the post intervention period. NMR: Decreased ~50% in the PHC village from the pre-intervention to the post-intervention period. No change in NMR in non-PHC villages. In PHC villages 65% of women were assisted at childbirth by a trained TBA during the post-implementation period and the proportion of women who delivered in a hospital or health centre increased.

Larsen et al 1983 [159]	South Africa. Rural community. Observational study. 4 traditional birth attendants caring for 22 pregnant women with no access to professional health care.	Assessed the impact of the training of TBAs over a 2 year period (intervention).	PMR: 0/1000. No statistical data given.

***Observational studies***			

Andersson 2000 [50]	Sweden (Sundsvall and Skelleftea). Population-based data Retrospective cohort study. Perinatal deaths (N = 4876) among N = 116211 newborns during the years 1831–1899.	Assessed the impact of the implementation of the midwifery system (43.7% of home deliveries were midwife assisted in 1871–1880 vs. 73.4% during the last decade of the century). Access to the midwives was 73.6% among more urban mothers vs. 50.8% among rural mothers.	PMR: 42/1000 births during the years 1831–1899. PMR: RR = 0.75 (95% CI: 0.66–0.84) among urban mothers comparing the decade after the midwifery system to the years before. PMR: RR = 0.79 (95% CI: 0.72–0.87) among rural mothers comparing the decade after the midwifery system to the years before. Prevented fractions of perinatal deaths: 32% vs. 15% comparing the decade after the midwifery system to the years before. respectively.

Egullion et al. 1985 [51]	Zimbabwe (Manicaland). Clinics, health centers and hospitals. An intervention study. Over 4000 TBAs were trained by December 1984.	To assess the impact on pregnancy outcomes of a culturally sensitive training of TBAs based on the risk approach and including information about clean delivery, pregnancy including ANC and birth preparedness, normal duration of labour, postnatal care, and harmful traditional practices. Training conducted by maternity nurses, and included fostering linkages between TBAs and the health system.	No statistical data provided, but "marked improvements" were noted, such as reduction in neonatal tetanus, and earlier arrival of obstructed labour cases at the hospital.

Kwast et al. 1996 [49]	Guatemala, Indonesia, Bolivia and Nigeria. Four different community-based projects between 1989 and 1993. Different study designs. Before-and-after studies in Guatemala and Indonesia.	Bolivia (the Warmi project): formed women's groups, trained birth attendants, husbands and women on safe birth practices, and strengthened referral linkages with the hospital, including a subsidy for hospital admissions. Guatemala (the Quetzaltenango maternal and neonatal health project): enhanced the skills of 400 TBAs vs. comparison areas where no such training was initiated. Indonesia (the Tanjungsari regionalization project): improved maternity services from village to hospital, including establishment of communication and transport links vs. a comparison sub-district without this intervention. Nigeria: provided life-saving skills training for midwives and interpersonal communication skills for all providers.	PMR (Bolivia): 105/1000 vs. 38/1000 births before and after the intervention, respectively. PMR (Guatemala): decreased among referred women in both the implementation and the control areas. 22.2% vs. 11.8% among referred women before and after implementation in the intervention area (P = 0.003). PMR (Indonesia): 47.7/1000 to 35.8/1000 births over 18 months of the project. 42.1/1000 vs. 25.9/1000 among all women delivered by the TBA during the last 6 months of the project. 98.7/1000 vs. 49.6/1000 among those with complications delivered by the TBA over the last 6 months of the project. Intrapartum SBR (Nigeria): 5.5% vs. 1.8% before and after the project. 57% reduction in postpartum haemorrhage (3.7 to 1.6%) and of 70% reduction in prolonged labour (from 20.6 to 6.2%).

Matthews et al. 1995 [52]	Nigeria (Uyo). Canadian-Nigerian safe motherhood project in the clan area within the catchment area of the government hospital. An intervention program. TBAs (N = 120) were registered for the course which took seven months.	To assess the impact of the training TBAs to use a pictorial method (a card with drawings and symbols) to identify and record risk conditions in childbirth on maternal and neonatal outcomes.	PMR: 25/1000 (20/795). Of these deaths, eight occurred in the group of mothers transferred to hospital. The remaining twelve babies had died at birth in the villages.

Foord 1995 [160]	Gambia (West Kiang district vs. Upper Baddibu district). An intervention study with a control district. N = 1516 women (794 in intervention and 722 in control area respectively)	To assess the impact of the intervention in the West Kian district through upgrading of personnel, TBA training, improved treatment and referral schemes, and increased number of visits to rural outreach areas vs. control district (Upper Baddibu) without this intervention.	Fetal death (miscarriage + SB): 39.9/1000 vs. 24.5/1000 in intervention and control districts, respectively. Early PMR: 54.9/1000 vs. 39.6/1000 in intervention and control districts, respectively.

* Web table could not be constructed because the review does not provide detailed data on studies with impact on perinatal mortality

Two of the projects in the multi-country MotherCare Project described by Kwast et al. [49] included components to improve TBA skills. In rural Guatemala, TBAs were trained to recognise and promptly refer pregnancy/delivery/neonatal complications, while the project simultaneously improved the quality of care in health facilities by modifying health professionals' attitudes towards TBAs and clients and implementing management protocols. In the intervention area, referrals from TBAs increased by 313% and perinatal mortality among referred women decreased from 22.2% to 11.8% (P = 0.003). In Indonesia, a project by the University of Padjadjaran sought to improve referral by TBAs and provide comprehensive essential obstetric care in the West Java subdistrict of Tanjunsari. Referrals to birthing centers by TBAs increased from 19% to 62%, and perinatal mortality declined from 47.7 to 35.8/1000 over 18 months ***[LOE: 2+]***.

Other observational studies have suggested directly or indirectly that TBA training can improve perinatal outcomes. Andersson et al. [50] conducted a before-after analysis of the introduction of a simple package of clean delivery ("antiseptic technique") practices, integrated into a package of improved neonatal care (e.g., provision of warmth, neonatal resuscitation with tactile stimulation for asphyxiated babies, cord care, and immediate breastfeeding) introduced long ago in Sweden by lay midwives in the late 1800, when perinatal mortality rates approximated those in many low-/middle-income countries today. They found that with the new practices, the prevented fraction of perinatal deaths increased from 15% to 32%  ***[LOE: 2+]***. In Manicaland, Zimbabwe, where 60% of births occur at home with relatives serving as birth attendants, Egullion [51] offered culturally sensitive training to 4000 TBAs and established linkages between TBAs and health facility staff. The hospital documented reduced neonatal tetanus cases and earlier arrival of obstructed labour cases at hospital, suggesting complicated labours were being more readily referred. No statistical significance data was given ***[LOE: 3]***. In Nigeria, as part of the Canadian-Nigerian Safe Motherhood Initiative, Matthews et al. [52] organised an pictorial education programme led by professional midwives to teach TBAs to recognise risk factors and improve their care of mothers, including completing antenatal cards during home visits. When tested after training, 70% of the TBAs correctly interpreted all of the 89 pictorial cards, and 89 of 110 TBAs had begun using the antenatal cards to monitor pregnancies. The project documented a perinatal mortality rate of 25/1000, but no baseline data were collected, precluding computation of impact ***[LOE: 3]***.

Two studies showed no or uncertain impact of TBA training on perinatal outcomes. In a rural area of The Gambia, Greenwood et al. [53] studied the impact of a primary health care (PHC) programme that included identification and training of a TBA in each village with a population of greater than 400. After the intervention, there were no differences in stillbirth rates in the intervention versus control villages (50/1000 vs. 51/1000) ***[LOE: 2+]***. In West Java, Indonesia, a longitudinal intervention study by Alisjahbana [54] implemented a comprehensive maternal health programme to improve maternal and perinatal health outcomes. The intervention area received training at all levels of the health care system (informal and formal) and birthing homes were established in villages; the control area received standard care. Over the entire study period, there was no statistically significant difference in perinatal mortality rates between intervention and control areas (43/1000 vs. 37/1000, respectively). Over time, perinatal mortality declined in the intervention villages (37/1000 vs. 50/1000 in Period 2 vs. Period 1, respectively) but not the control villages, but whether this decline was statistically significant was not reported ***[LOE: 2+]***.

##### Conclusion

The potential of TBA training to reduce perinatal mortality is promising, especially when TBA care is integrated with quality health services or health services strengthening activities. An example is the reductions in perinatal and possibly maternal deaths observed in rural Pakistan where home births are the norm, but where TBAs, women and families had access to an improved health system with a clinical outreach component [47]. A number of other studies [49,51,53] we reviewed also reported promising results for TBA training. Challenges and controversies surround the best methods to train TBAs in techniques for clean delivery and recognition and effective referral of complications. To be successful, such training strategies must take into consideration the best candidates for training (e.g., type of birth attendant, number of deliveries/year, willingness to be trained and to refer complications); the need for refresher training and ongoing supervision; the need to link them with the health system for management of complications; the possible need for inputs such as basic resuscitative equipment or supplies for clean delivery; possible tensions between TBAs and the formal health system that can complicate or discourage referral; and compensation or incentive strategies for TBAs who refer women to health facilities.

#### Training of other cadres of Community Health Workers (CHWs)

##### Background

In addition to TBAs and nursing cadres including nurse-midwives and nurse-aides, CHWs are active in the health systems of many rural or underserved settings. These individuals may include current or former personnel associated with projects of non-governmental organisations, paid or unpaid participants in government health promotion or education schemes, and volunteers. With training, CHWs can function as community activists, opinion leaders, or health promoters, and can share their knowledge with community members, including pregnant women and their families. Because the availability, abilities, and prior training of CHWs vary significantly from setting to setting, relatively few have been broadly integrated with public sector programmes and health systems to promote activities that could prevent stillbirth, although there is increasing focus on the use of CHWs to provide postnatal care for mothers and newborns.

##### Literature-based evidence

We identified 1 Cochrane review comprised of 11 RCTs, as well as 2 other observational studies (Table 3). Hodnett et al. [55] conducted a systematic review of 11 trials of additional social support versus usual care for poor pregnant women at risk of low birth weight (LBW). There was a non-significant effect of such support on stillbirth/neonatal death (RR = 1.15, 95% CI: 0.89–1.51) ***[LOE: 1+] ***(Additional file 2).

**Table 3:** Impact of training community health workers on stillbirths and perinatal mortality

**Source**	**Location and Type of Study**	**Intervention**	**Stillbirths/Perinatal Outcomes**
***Reviews and meta-analyses***			

Hodnett et al. 2003 [55]	USA, South Africa, England, Rosario, Argentina; Pelotas, Brazil; Havana, Cuba; and Mexico City. Meta-analysis (Cochrane). 5 RCTs included (N = 9507 poor women)	Assessed the effects of additional antenatal support (intervention) vs. usual care (controls) during pregnancies at risk of low birth weight.	SBR/NMR: RR = 1.15 (95% CI: 0.89, 1.51) **[NS]**. [112/4778 vs. 96/4729 in intervention and control groups, respectively].

***Intervention studies***			

Bhutta et al. 2008 [56]	Pakistan (Sindh). Rural community (8 village clusters). Pilot study. Before-after intervention data in both intervention and control villages. N = 3747 pregnant women (N = 2056 in the intervention villages, N = 1691 in the controls).	Compared the impact of an intervention where Lady Health Workers (LHWs) and TBAs (Dais) received enhanced training in newborn care and established close liaisons with each other as well as community mobilization activities (intervention) vs. control villages where the regular LHW training programme was continued, but no attempt was made to link LHWs with the Dais.	SBR: RR = 0.66 (95% CI: 0.53–0.83); P < 0.001. [65.9/1000 vs. 43.1/1000 births before and after the intervention in intervention villages, respectively]. SBR: RR = 1.04 (95% CI: 0.84–1.30); P = 0.23 **[NS]**. [58.1/1000 vs. 60.5/1000 births before and after the intervention period in the control villages, respectively]. PMR: 110.8/1000 vs. 72.5/1000 before and after the intervention in intervention villages, respectively. PMR: 94.64/1000 vs. 101.2/1000 before and after the intervention period in the control villages, respectively.

Mercer et al. 2004 [57]	Bangladesh. Rural community. Before-after study design. N = 27 partner NGOs from 1996–2002 funded by the Bangladesh Population and Health Consortium.	To assess the effectiveness of a non-governmental organization (NGO) primary health care programme utilising female Family Health Visitors (FHV) who were responsible for basic health and family planning counseling, doorstep delivery of contraceptives and oral rehydration salts, and mobilization of women to use satellite clinics and higher level facilities.	NMR: 39.0/1000 in 1996 (baseline). From 1999–2002, decreased consistently from 36.8 to 15.1/1000 live births among the poorest, and from 30.6 to 16.5/1000 among the remainder of women.

As part of a larger cluster RCT, a pilot study in rural Pakistan by Bhutta et al. [56] encouraged community health committees to work with volunteer local CHWs called Lady Health Workers and TBAs called *dais *to provide domiciliary care and health education. In the intervention villages, Lady Health Workers worked in conjunction with *dais *and both cadres were trained in enhanced neonatal care. Stillbirth rates declined significantly in intervention villages (from 66 to 43/1000) as did early neonatal mortality rates (from 48 to 31/1000). Declines in control areas served by Lady Health Workers without this neonatal care training and without liaisons with *dais *were not significant. Skilled birth attendance increased in public sector facilities (34% versus 20% at baseline) and home births in the intervention villages correspondingly declined (65% versus 79% at baseline) ***[LOE: 1+]***.

Another intervention study by Mercer et al. [57] in rural Bangladesh used Family Health Visitors as part of a primary health care programme to promote basic health and family planning as well as mobilise women to use formal health care facilities for antenatal and obstetric care. From 1999–2002, the neonatal mortality rate decreased consistently from 36.8 to 15.1/1000 among the poorest women, and from 30.6 to 16.5/1000 among the remainder of women ***[LOE: 2-]***.

**Figure 2:**
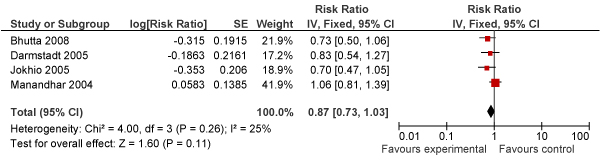
**Meta-view (Forest plot) of the impact of community-based intervention packages on stillbirths**.

A pooled analysis in the Lancet Primary Healthcare Alma Ata Series [7], based on three large cluster RCTs, showed a 29% reduction in the risk of perinatal mortality with a package of community based interventions including health promotion by CHWs (3 studies; RR = 0.71; 95% CI: 0.61–0.84 ([47,56,58]. These interventions were frequently based on packages of promotional and preventive services through a range of CHWs working in close liaison with TBAs and community representatives. The potential pathways for impact are complex and in the case of Pakistan [56], India [58], and Nepal  [60] possibly operated through a combination of improved domiciliary practices and increased skilled attendance. Notwithstanding these variations in approaches, a preliminary meta-analysis of recent studies (Figure 2) indicates a 13% reduction in stillbirths attributable to these packages (RR = 0.87; 95% CI: 0.73–1.03) [47,56,59,60].

##### Conclusion

Despite considerable interest in the field, and increasing evidence for neonatal and maternal mortality reduction there is a paucity of data from studies on the effects of CHWs' activities on stillbirth outcomes. While the available studies provide some evidence of the benefit of utilizing CHWs on reduction of stillbirths, there is a need for further research, especially large-scale studies to evaluate the role of alternative cadres of community workers in reducing or preventing adverse pregnancy outcomes.

#### Training nurse-aides (including task-shifting) as birth attendants

##### Background

In many low- and middle-income countries, particularly in rural areas, the most common cadre of health worker is the nurse-aide (also referred to as nursing aide, nursing assistant, auxiliary nurse/nurse-midwife, or nursing associate). Some initiatives have offered training to equip nurse-aides to deliver a broad spectrum of preventive and curative health care services. Because of potential cost savings and availability of nurse-aides, especially in rural settings, there is an interest in task shifting to nurse-aides or similar cadres in geographic areas with insufficient physicians and nurses to manage all deliveries.

##### Literature-based evidence

We identified one observational study on the role of nurse-aides (Table 4). Manungo et al. [61] described perinatal mortality rates associated with nurse-aides conducting low-risk deliveries at a mission hospital in rural Zimbabwe. The study reported very low perinatal mortality among births assisted by nurse-aides (5/1000), who attended 57% of births. Doctors and nurses at the hospital handled births of primigravidae and high-risk pregnancies, which had substantially higher perinatal mortality (57/1000 births). While perinatal mortality rates are not comparable because of differences in risk profiles between the two groups, the study suggested that risk screening was effective and that nurse-aides' skills were sufficient to attend low-risk births in this setting ***[LOE: 2-]***.

**Table 4:** Impact of training nurse-aides as birth attendants on stillbirths and perinatal mortality

**Source**	**Location and Type of Study**	**Intervention**	**Stillbirths/Perinatal Outcomes**
***Observational studies***			

Manungo et al. 1996 [61]	Zimbabwe. Rural hospital. Retrospective analysis of records. N = 1459 deliveries, of which N = 824 (57%) were conducted by nurse aides.	Compared the impact of the nurse-aide-conducted deliveries (exposed) vs. those by the trained medical staff (unexposed) on perinatal mortality.	SBR: 1/824 vs. 13/635 in the nurse aide vs. trained staff groups, respectively. PMR: 5/1000 vs. 57/1000 births in the exposed vs. non-exposed groups, respectively.

##### Conclusion

The perinatal death rate among low-risk deliveries conducted by nurse-aides in the study from Zimbabwe was relatively low; however, additional data is needed, especially on the performance of nurse-aides in circumstances where referral is difficult and whether nurse-aide cadres could be trained to manage relatively complicated deliveries. We have classified the overall evidence of this intervention as Grade D and underscore the need for robust evaluation of such interventions in various circumstances before any conclusions can be made.

#### Training to improve skills of professional midwives in antenatal and intrapartum care

##### Background

In low-risk pregnancies, evidence suggests that antenatal and intrapartum care can be managed effectively by providers other than obstetricians. Midwives have long attended births, predating the field of obstetrics by many years. Before modern obstetrics, professional midwifery practice brought about significant reductions in perinatal mortality, as in northern Sweden in the 1800s [50]. In modern practice, the term "midwife" refers to different groups of individuals with vastly different training levels, ranging from apprenticeship with no formal training (lay midwives; referred to as TBAs in this review) or a few weeks of biomedical training (trained TBAs) to individuals with nursing degrees and graduate-level midwifery training (certified nurse-midwives). Midwives with significant formal midwifery training (at least one year) and who function as part of the formal health system are referred to in this paper as "professional midwives," to distinguish them from TBAs and midwives with less training. Professional midwives routinely provide antenatal care and health education, and have the requisite technical skills to provide safe birthing services for uncomplicated deliveries, and to recognize and refer patients to obstetricians or other specialists in cases of complications. Their approach is generally holistic, culturally sensitive, and centred on the preferences of the women in their care [62]. This section reviews the evidence for their impact on stillbirths.

##### Literature-based evidence

The review of literature identified 3 Cochrane reviews comprised of 15 RCTs and 3 other intervention, quasi-experimental and observational studies (Table 5). Comparing trials of midwife- or general-practitioner managed care versus obstetrician-gynecologist led shared care, Villar et al. [63] found a non-significant reduction in perinatal mortality among births managed by midwives compared to those where physicians and midwives shared care for the parturient (OR = 0.59, 95% CI: 0.28–1.26) ***[LOE: 1++] ***(Additional file 3).

**Table 5:** Impact of training professional midwives on stillbirths and perinatal mortality

**Source**	**Location and Type of Study**	**Intervention**	**Stillbirths/Perinatal Outcomes**
***Reviews and meta-analyses***			

Hodnett 2000 [161]	UK (London) and Australia (New South Wales). Meta-analysis (Cochrane). 2 RCTs included (N = 1815 participants).	Assessed the effects of antenatal, intrapartum and postpartum care by midwives (intervention) vs. usual care by multiple caregivers (controls).	SBR/NMR: OR = 1.96 (95% CI:: 0.83–4.63) **[NS]**. [14/908 vs. 7/907 in intervention and control groups, respectively].

Hodnett et al. 2003 [55]	Australia, US (South Carolina), UK and France. Meta-analysis (Cochrane). 11 RCTs included (N = 9507 participants).	Assessed the effects of social support from midwives (intervention) vs. usual care (controls) antenatally for pregnancies at risk of low birth weight.	SBR/NMR: RR = 1.15 (95% CI: 0.89–1.51) **[NS]**. [112/4778 vs. 96/4729 in intervention and control groups, respectively].

Villar et al. 2001 [162]	UK and Scotland. Meta-analysis (Cochrane). 2 RCTs included (N = 2890 low-risk women).	Assessed the effects of midwife/general practitioner managed care (intervention) vs. obstetrician/gynecologist led shared care with midwives (controls) on perinatal mortality.	PMR: OR = 0.59 (95% CI: 0.28–1.26) **[NS]**. [10/1447 vs. 17/1443 in intervention and control groups, respectively].

***Intervention studies***			

Ibrahim et al. 1992 [66]	Sudan. Community-based (48 rural villages). Prospective before-after study spanning three years (March 1985–April 1988). N = 6275 deliveries monitored by 40 village midwives, of which 150 were stillbirths and 167 neonatal deaths.	Assessed the impact of training and upgrading of the skills of village midwives (licensed, with 1 year of midwifery training) starting from the middle of the second year of the study.	SBR: 43/1845 (2.3%) vs. 55/2132 (2.6%) vs. 48/2298 (2.1%) in 1985–86, 1986–87 and 1987–88 respectively. SBR+NMR: RR = 0.75 in the third year in comparison with the first two (P < 0.05).

Theron et al. 2000 [64]	South Africa (Eastern Cape Province). Prospective, controlled trial. N = 73 midwives (N = 34 in the study town, N = 39 in the controls).	The practical skills of midwives caring for pregnant women were determined before and after the introduction of training via Maternal Care Manual in the study town. No training was given in the control towns.	Distribution of marks: a significant (P < 0.001) improvement occurred in the study town between pre- and postintervention periods, whereas the control towns showed no change. The mean improvement in the study town was 3.5 marks (36.6% improvement) vs. 0.1 marks (1.1% improvement) in the control towns.

***Observational studies***			

Montero-Mendoza et al. 2000 [163]	Mexico (Chiapas). Rural and urban area. Cross-sectional study. N = 670 women between 15 to 49 years old with N = 1,382 pregnancies from 1987 to 1996.	Compared the impact of birth assistance by a midwife (study group) vs. a relative of the pregnant woman, her husband or herself (controls).	PMR: OR = 0.30 in study vs. control groups, P < 0.01. (36.2/1000 live births)

A Cochrane review by Hodnett ED et al. [62] reviewed continuity of care by caregivers, which is a hallmark of the midwifery model of intrapartum care (16 trials, N = 13,931 women) (Additional file 4). Continuous intrapartum support was associated with shorter labour, more spontaneous vaginal births and less need for intrapartum analgesia. Although the risk of stillbirth/neonatal death was non-significantly increased (OR = 1.96, 95% CI = 0.83–4.03), the likelihood of Caesarean section was lower (RR = 0.91, 95% CI: 0.89–0.99) ***[LOE: 1++]***. Another Cochrane review [55] found no difference in stillbirths or neonatal deaths when midwives provided antenatal social support to economically disadvantaged pregnant women at risk of low birth weight compared to controls who did not receive this support (RR = 1.15, 95% CI: 0.89–1.51 [NS]) (Additional file 2).

In South Africa, Theron et al. [64] conducted a prospective, controlled trial assessing the practical skills of midwives after completion of a distance-learning self-education tool called the Perinatal Education Programme Maternal Care Manual. [65] Pre- and post-tests during midwifery practical skills assessment showed improvement of 36.6% among midwives who studied the manual ***[LOE: 2+]***. In rural Sudan, a prospective study by Ibrahim et al. [66] (N = 6275 deliveries) introduced a program to upgrade the skills of village midwives during the second of the three study years, observing a 25% reduction in the risk of stillbirth/neonatal death in the third year relative to the first 2 years. As stillbirth rates over the three years were relatively similar, most of this reduction reflects improved newborn survival ***[LOE: 2-]***.

Fauveau et al. [67,68] evaluated a community-based program in Matlab, Bangladesh, which included training midwives posted to study villages who were asked to attend as many home deliveries as possible, detect and manage complications, and accompany women with complications to the project central maternity clinic. Maternal deaths and perinatal mortality significantly declined in the intervention areas compared to control populations. In the same study area, Ronsmans et al. [69] reported a stillbirth rate reduction in the intervention area of 24% (crude OR comparing post-project with pre-project rates = 0.76; 95% CI: 0.68–0.84), compared with a 15% reduction in the government (control) service area (crude OR comparing post-project with pre-project rates = 0.85; 95% CI: 0.76–0.94); the pace of decline in the intervention area was statistically significantly more rapid than in control areas (P-value for adjusted time × area interactions for stillbirth = 0.023).

Bergstrom and colleagues have published a series of evaluations of the use of non-physician attendants to perform Caesarean sections. In a pilot study in Mozambique, medical surgical assistants were trained to provide Caesarean sections in rural hospitals [70]; post-operative complication rates were comparable to Caesarean sections performed by obstetricians or gynecologists [71]. Subsequent evaluations in Tanzania and Malawi have established that non-physician attendants provide most of the Caesarean sections, are lower-cost to train, have higher retention with no measured difference in complications or infection rates. [72-74]

##### Conclusion

There is some evidence that midwifery training programs leading to improved midwifery skills can reduce intrapartum complications and perinatal outcomes, including reduction in stillbirth incidence. Improvements in practical obstetric skills of midwives followed training, and midwives appeared to manage low-risk births without increasing, and possibly reducing, rates of perinatal mortality. Technical skills in providing continuous care during childbirth may be more influential on birth outcomes than provision of antenatal social support. The evidence consists primarily of observational data; large-scale studies with appropriate designs are thus needed to evaluate the potential impact of trained midwives on stillbirth and perinatal mortality rates. In addition, the midwives' retention of the training must be measured longitudinally in larger studies.

#### Obstetric drills

##### Background

Obstetric drills are increasingly used as a means to test provider skills, improve and maintain provider knowledge, and ensure competency and efficiency of staff, particularly in health facilities where life-threatening emergencies are rarely seen and skills may deteriorate [75]. In the UK, a survey of obstetric emergency drills showed that half of the centres surveyed already conducted drills, and an additional 14% had a drill programme under development [76]. Drills have been shown to positively impact physician practices when using standardised technical manoeuvres and checklists [77], and accordingly, both the American College of Obstetricians and Gynaecologists (ACOG) and the U.S. Joint Commission for Patient Safety Standards have recommended obstetric drills for shoulder dystocia, neonatal resuscitation, Caesarean section, and maternal haemorrhage [78,79]. Drills have also been used for management of eclampsia and other obstetric complications and procedures.

For a given obstetric emergency, a drill generally involves an algorithm specifying the actions of each provider on the team, a clinical plan of action to manage the complication, and an outline to ensure appropriate documentation and follow-up [80]. Drills may be videotaped or a scribe may be present, offering an opportunity for the medical team to review the drill more objectively in retrospect to identify areas for improvement [81].

Drills may occur in a real-world environment on the maternity ward or in the emergency room, with a local team of providers. The equipment, psychological reality, and team dynamics are the same as participants' experience on the job, which distinguishes drills from other training and performance improvement strategies which rely on classroom-based, computer-driven simulations. In simulations, participants are sent out for training, which often revolves around the use of high-tech mannequins [82,83]. Simulations, however, have shown some evidence of improved technique. Simulations for shoulder dystocia by the Simulation and Fire-drill Evaluation (SaFE) trial in the UK demonstrated improved management by providers that was largely retained a year later [83,84]. In contrast to these simulation studies, few studies have reported the impact of in-hospital drills – a lower-tech, real-world exercise – on provider practice or outcomes. By improving coordination between providers, reducing delays and errors, and remedying deficiencies in the technical interventions provided, obstetric drills could plausibly have an impact on stillbirths.

##### Literature-based evidence

We identified four observational studies reporting the implementation of obstetric drills. None reported stillbirths or other perinatal outcomes.

In Beirut, Lebanon, Osman et al [75] conducted a prospective trial of emergency obstetric drills at 3 hospitals, including 2 tertiary facilities and a community hospital. At each facility, 2 drills were conducted, recorded, and reviewed critically at 2 different points during April and May 2006. Drills were conducted either on the labour ward or in the emergency room, and employed an actor posing as a pregnant woman with a research assistant posing as her companion, and on-duty medical and paramedical staff including an obstetrician (the drill leader). While overall quality of care was within acceptable standards of care, the exercise unearthed problems associated with supplies and equipment, hospital policy, and clinical handling of the emergency ***[LOE: 2-]***.

In a hospital in Wisconsin, USA, Curtis et al. [80] described the development and implementation of an emergency obstetric drill focused on nursing cadres, specifically for shoulder dystocia. The drill included a video sensitizing nursing staff to the signs of and management of dystocia, an algorithm which directed defined roles for all team members, and an acronym to help participants remember the plan of action. The drill stressed the need for careful coordination and good communication on the team, and included careful review of recorded video by all participants after the drill, as well as a survey to evaluate attitudes toward the drill. Drills were attended by 98% of nursing staff, 80% of obstetricians/gynecologists, and 57% of family practitioners conducting deliveries at the hospital. In addition to neonatal resuscitation drills, which are used in conjunction with the shoulder dystocia drills, the hospital is developing drills for emergency Caesarean section and maternal haemorrhage based on the success of the shoulder dystocia drills ***[LOE: 1-]***.

In 6 hospitals in Minnesota, USA, Miller et al. [85] conducted a pilot study of 35 *in situ *obstetric emergency drills requiring emergency Caesarean section (for placental abruption, ruptured uterus) and management of postpartum haemorrhage involving physicians, nurses, and support staff (N = 700; N = ~20 per simulation). An actor played the pregnant woman, and fetal mannequins in plastic fluid-filled "uteruses" were used. A physician and nurse team created scenarios including sudden clinical changes and distractions to create stress to test the participants. Following the drill, each team was debriefed for 2 hours to share lessons learned about communication, teamwork, and safety. The drills effectively elicited failures in teamwork that have led to a new focus on team-building within the hospital system ***[LOE: 2-].***

In a tertiary referral unit in Sydney, Australia, Thompson [86] reported the results of a programme involving on-site simulation of patients with eclampsia to test emergency systems for handling eclampsia. Staff suffered from inexperience because eclampsia was rare on the maternity ward. The drills resulted in rapid activation of the emergency team, development and dissemination of an evidence-based protocol for eclampsia, and the strategic placement of "eclampsia boxes," as well as efficient and appropriate management of subsequent simulated patients ***[LOE: 1-]***.

##### Conclusion

Obstetric drills, primarily in high-resource settings, have been shown to be a useful team-oriented tool to identify and address deficiencies including provider error in emergency obstetric care in health facilities. Staff in multiple settings has found them to be acceptable and helpful. Drills and effective simulations have been developed and implemented for shoulder dystocia management, but there are few for more common complications/procedures associated with poor perinatal outcomes, such as emergency Caesarean section. Obstetric drills could lead to improved quality of care for obstetric patients, as well as reductions in adverse perinatal outcomes including stillbirth [87], but there are not yet any studies that have measured these outcomes (Grade C evidence). We encourage the collection of perinatal outcome data subsequent to implementation of obstetric drills and other emergency obstetric training measures for performance improvement.

#### Training in neonatal resuscitation for physicians and other health care workers

##### Background

Many intrapartum stillbirths as well as neonatal deaths are associated with acute intrapartum events such as cord accidents, haemorrhage, hypertension, or prolonged or obstructed labour. Some babies that appear to be stillborn at birth may be able to be resuscitated if immediate and appropriate resuscitation techniques are used; though these are technically neonatal deaths, they often are documented as stillbirths, especially in low resource settings lacking in diagnostic tools and technologies. As an example, Airede et al. [88] conducted an audit of perinatal deaths at one hospital in Nigeria and implicated lack of or delayed resuscitation at birth in 46.2% of these deaths. Appropriate resuscitation skills are thus potentially important in reducing rates of early neonatal deaths which are often mis-classified as stillbirth. While ANC can identify fetal distress as well as risk factors for birth asphyxia, a significant proportion of babies who will require resuscitation at birth cannot be identified antenatally. It is thus important that all personnel involved in labour room care of the newborn should be fully trained in neonatal resuscitation.

##### Literature-based evidence

Our literature search identified seven intervention or observational studies examining programs that trained health professionals to provide neonatal resuscitation (Table 6). Several intervention studies have examined the impact of hospital-wide or nation-wide neonatal resuscitation programs on pregnancy outcome [89-95]. In a Chinese study by Zhu et al [91], the introduction of a neonatal resuscitation programme resulted in a 3-fold reduction in early neonatal mortality (χ^2 ^= 10.54, P < 0.01) ***[**LOE: 2-**]***, and in India, introduction of a neonatal resuscitation programme in 14 teaching hospitals [90] increased awareness and documentation of birth asphyxia, associated with a significant decline in asphyxia-related deaths (P < 0.01) ***[******LOE: 2-]***. Another study of a basic neonatal resuscitation programme in rural India by Cowles [89] which trained nurses and ward aides reported decreased rates of stillbirths compared to hospitals where the resuscitation course was not offered.

**Table 6:** Effect of training in neonatal resuscitation for physicians and other health workers on stillbirths and perinatal mortality

**Source**	**Location and Type of Study**	**Intervention**	**Stillbirths/Perinatal Outcomes**
***Intervention studies***			

Cowles 2007 [89]	Northern India. Rural hospitals. An intervention study.	Assessed the impact of the Basic Neonatal Resuscitation Program (BNRP) for the birth attendants (nurses and ward aides) in providing more effective neonatal resuscitation at birth.	SBR: decreased in the hospitals where the course had been taught on site. Doctors stated that, when called for resuscitation, they would find the nurses giving the Ambu Bag, and a living baby, when before the babies had died.

Deorari et al. 2001 [90]	India. 14 teaching hospitals. Before-after study. N = 28 faculty members from each hospital, who in turn trained staff at their own hospital. Each institution provided 3 months pre-intervention and 12 months post-intervention data.	Compared the impact for 12 months after (intervention) vs. for 3 months before (control) use of Neonatal Resuscitation Programme (NRP) in teaching hospitals to doctors and nurses.	Total cause-specific NMR: 901/25,713 (3.5%) vs. 264/7,070 (3.7%) after and before the intervention, respectively. (P > 0.05). Asphyxia-related cause-specific mortality: Significant reduction (P < 0.01).

Jeffery et al. 2004 [164]	Macedonia. 16 participating hospitals. National perinatal strategy programme.	Assessed the impact of a train-the-teachers education intervention to develop the capacity of health professionals to introduce evidence-based perinatal practice originally developed in Australia.	PMR: RR = 0.79 (95% CI: 0.73–0.85). [21.5/1000 vs. 27.4/1000 after vs. before, respectively]. Early NMR: 36% reduction (infants > 1000 g birth weight) A total of 115 doctors and nurses graduated from this programme.

Kumar et al. 1994, 1995 [98,97]	India. Rural setting. Prospective cohort study. N = 58 cases of asphyxia; 38 delivered by conventionally trained TBAs [N = 968] and 20 by TBAs with advanced training [N = 911].	Simplified methods of resuscitation were taught to TBAs. An additional group received advanced training on use of the mucous extractor and bag-and-mask ventilation.	PMR: 19% reduction comparing advanced vs. conventionally trained TBAs, respectively. Asphyxia-associated perinatal mortality: 70% reduction comparing advanced vs. conventionally trained TBAs, respectively.

Raina et al. 1989 [96]	India (Haryana). Villages of Ambala District. Exploratory study. TBAs (N = 100) where 90% of deliveries occur at home, and are performed by TBAs.	To assess the training needs of traditional birth attendants with reference to their knowledge of the causes of birth asphyxia, their capacity to recognise it and the methods they were using to manage the condition.	TBAs mentioned 6 resuscitation measures they used in birth asphyxia. 4 or more of these were only used by 20 of the TBAs. 70% of the participants used resuscitation procedures for 1/2 hour before giving up due to such prognostic features as a blue or pale color, the absence of cord pulsations, no breathing, limpness and the absence of pulsations in the anterior fontanelle. Knowledge of modern resuscitation equipment and procedures was poor and referrals were made based on the proximity of the institution and not on the quality of care available.

Zhu et al. 1997 [91]	China (Zhuhai). Maternal and Child Health Hospital. Perspective, before-and-after intervention study. N = 4,751 newborns with 366 asphyxiated babies in a period of 2 years and historical controls of 1,722 live births.	Compared the impact on neonatal mortality of the Neonatal Resuscitation Program (NRPG) (intervention) vs. historical controls when the traditional resuscitation program was in place.	PMR: 3-fold decrease with NRPG compared to historical controls (chi(2) = 10.54, P < 0.01).

***Observational studies***			

Blond et al. 1994 [93]	France. 31 maternity hospitals. Before-after study. N = 156 medical personnel (doctors, mid-wives) and paramedics [53% of all available personnel].	To assess the impact of special training to medical personnel (intervention) vs. personnel without any special training (control) in 1990.	Improved neonatal resuscitation rates compared with untrained personnel. Severe meconium aspirations: 0 vs. 3 in 1990 vs. 1989, respectively.

A more controversial yet urgent issue concerns interventions to resuscitate asphyxiated newborns born at home in the absence of skilled attendance. Raina et al. [96] reported that TBAs in Haryana, India, were readily able to recognise birth asphyxia, but lacked modern resuscitative knowledge and skills. TBAs were found to use 6 different resuscitative techniques, but only 20% of the sample used more than 4 of these techniques, which were not assessed for their effectiveness. Efforts of TBAs to resuscitate newborns suggest that if they could be trained in resuscitative techniques and given basic equipment, perinatal deaths might be reduced ***[LOE: 3]***. In rural India, Kumar et al. [97,98] reported that asphyxia-associated perinatal mortality was 70% lower [P < 0.05] among babies delivered by traditional birth attendants trained to perform resuscitation using a mucous extractor and bag-and-mask resuscitation device (advanced resuscitation) versus simplified resuscitative training. Overall perinatal mortality was 20% lower in the group of asphyxiated infants delivered by TBAs with advanced training compared with simplified training, but the sample size was small and the finding was not statistically significant ***[LOE: 2-]***.

##### Conclusion

There are only a few studies examining the impact on stillbirths/perinatal mortality of training health professionals or other individuals to perform neonatal resuscitation. One study reported a statistically significant decrease in perinatal mortality [91], while in the other study there was a decrease only in asphyxia-related deaths [90]. There is some evidence of reduction in stillbirths and perinatal mortality after training health workers in resuscitation skills, but further evidence is needed from rigorous, ethically designed and controlled studies. Such studies should measure the impact of programs to improve health care providers' resuscitation skills, including whether individuals with minimal training such as TBAs can perform resuscitation safely and effectively. Additionally, there is a need for more general studies of stillbirth incidence in hospitals with differing policies and capacity to resuscitate asphyxiated newborns.

### Health system organizational strategies

#### Public-private partnerships to provide emergency obstetric care

##### Background

In low-/middle-income countries, cost and distance are major barriers to care-seeking, particularly in cases of obstetric complications. In many rural and under-resourced areas, particularly at the district health system level, there is a dearth of skilled care providers practicing within the public health system. In many of these areas, private sector facilities and practitioners that provide comprehensive essential obstetric care exist, often providing higher-quality services than public sector services, but the poorest women often cannot afford the fees, and thus cannot access, these services [99]. Public-private partnerships offer one potential solution. Such partnerships vary widely in structure and function, and can range in size and complexity from small collaborations with industry or mission hospitals to large collaborative efforts between governments and private NGOs or UN agencies. In public-private schemes, public funds may be used to fund the cost of private providers' services to strengthen health services. New or expanded provider networks, often with district health official input, improve coverage at low or no cost to the rural poor. There are many different types of public-private partnerships, many of which involve community partnerships with a broad range of civil society groups and health care professionals to galvanise communities and health systems for perinatal health [100]. Few public-private partnerships have addressed the provision of antenatal and/or obstetric care, or comprehensive essential obstetric care, and few have assessed birth outcomes in relation to changes in the system of care.

##### Literature-based evidence

A literature review identified one observational study from India that reported stillbirths or perinatal mortality associated with activities of a public-private partnership (Table 7). In southern India, after the creation of a special care neonatal unit at a district government hospital using private funding and NGO support, Shantharam Baliga et al (2007) [101] reported that antenatal referrals from community health centres increased 48.6% and neonatal admissions increased 14.7%. These increased referrals coincided with reduced rates of hospital stillbirths (35.5 vs. 44.8/1000 births, after vs. before) and perinatal deaths (50.2 vs. 65.8/1000 births, after vs. before) [99,100].

**Table 7:** Impact of public-private partnerships for providing comprehensive essential obstetric care on stillbirths and perinatal mortality

**Source**	**Location and Type of Study**	**Intervention**	**Stillbirths/Perinatal Outcomes**
***Observational studies***			

Shantharam Baliga et al. 2007 [101]	India. Government District Headquarters Maternity Hospital. Before-after intervention study.	Tracked the impact of scale-up of neonatal services from 1998–2001, and compared outcomes in 2004 (post-upgrade) to 1998 (pre-upgrade)	Hospital SBR: 44.79 vs. 35.52/1000 live births in 1998 and 2004, respectively; P = 0.04. Hospital PMR: 65.81 vs. 50.15/1000 live births in 1998 and 2004, respectively; P = 0.003. Hospital early NMR: 21.02 vs. 14.63/1000 live births in 1998 and 2004, respectively; P = 0.03.

##### Conclusion

These preliminary data suggest that participatory provision of services through public-private partnerships in district health systems could improve maternal and perinatal outcomes. Very few studies, however, have been conducted to evaluate the impact of public-private partnerships to increase access to emergency obstetric care. Factors important to sustainable delivery of care include an enabling environment, assured payment mechanisms for providers, and good collaboration and communication between public and private partners [102]. Projects are underway to reduce maternal and neonatal mortality through public-private partnerships to finance private provider care in rural areas, such as the Chiranjeevi Project in Gujarat State, India, but no impact data on stillbirths are available [103]. Further well-designed interventions and evaluations are needed to evaluate the cost-effectiveness and sustainability of these approaches in efforts to prevent stillbirth in low-/middle-income countries.

#### Maternity waiting homes

##### Background

In low-/middle-income countries, the distance and time required to reach health facilities are often obstacles to care-seeking. Financial constraints may also impact a woman's ability to obtain transport to a hospital and these delays contribute to poor birth outcomes among those families with the least resources. Maternity waiting homes – lodgings for pregnant women close to or within hospitals – are a strategy to address these access barriers. Provision of rapid transfer to hospital for women with high-risk pregnancies is another strategy. Waiting homes have been recommended by the World Health Organization to reduce maternal morbidity and mortality [104], but the evidence for impact on stillbirths and neonatal outcomes has not been systematically summarised.

##### Literature-based evidence

Our literature search identified 5 intervention/observational studies assessing the impact of maternity waiting homes on birth outcomes (Table 8). In rural Zambia, Van Lonkhuijzen et al. [105] compared prevalence of pregnancy risk factors (classified as maternal or antenatal) and pregnancy outcomes among women staying at a maternity waiting home versus women who gave birth in a hospital after direct admission. The prevalence of risk factors was statistically significantly higher among waiting home users than among women who were directly admitted to the hospital (83% vs. 53% had at least one maternal risk factor, respectively; and 22% vs. 15% had at least one antenatal risk factor, respectively). Maternal and perinatal mortality rates were comparable between the two groups, but it is plausible that the waiting home use reduced maternal and antenatal mortality among the higher-risk women served by the maternity waiting home to the same levels as the lower-risk women who went directly to hospital ***[LOE 2-]***.

**Table 8:** Impact of maternity waiting homes on stillbirths and perinatal mortality

**Source**	**Location and Type of Study**	**Intervention**	**Stillbirths/Perinatal Outcomes**
***Intervention studies***			

Guruvare et al. 2007 [109]	India. Six satellite maternity homes attached to the tertiary care hospital. Descriptive intervention study.	To assess the perinatal mortality rate among pregnant women taken care of at the hospital, along with the attached satellite maternity homes. This rate was compared with the national average.	PMR: 21/1000 vs. 70/1000 live births in the study group vs. the national average. (63% of perinatal deaths were stillbirths).

***Observational studies***			

Chandramohan et al. 1995 [106]	Zimbabwe. Rural hospital-based. Cohort study. N = 4488 high risk pregnant women (N = 1573 in the intervention group, N = 2915 in the controls) during the period 1989–1991. Information on antenatal risk factors, use of ANC, access to the hospital and stage of labour on arrival was collected for each woman.	Compared the effect of staying in a maternity waiting home from 36 wks to delivery (intervention) vs. going straight from home to hospital at the time of delivery (controls).	SBR: 19.2/1000 vs. 10.8/1000 in the control and intervention groups, respectively. PMR: RR = 1.7 (95% CI: 1.1–2.6); P < 0.05. [32.2 vs. 19.1/1000 in the control vs. intervention groups, respectively]. PMR: adj. RR = 1.5 (95% CI: 0.95–2.5); P = 0.07 in the control vs. intervention group. However, when the analysis was restricted to women with antenatal risk factors there was a significant 50% reduction in the risk of perinatal death for the women in the intervention vs. controls (adj. RR = 1.9; 95% CI: 1.1–3.4; P < 0.05).

Poovan et al. 1990 [108]	Ethiopia. Hospital based. Prospective cohort study. N = 777 pregnant women at high risk of complications or those living in remote areas (N = 142 intervention group, N = 635 controls).	Compared the impact of either coming via a maternity waiting home (intervention) vs. coming directly to the hospital (controls).	SBR: 28.2/1000 (4/142) vs. 253.5/1000 (161/635) births in intervention and control groups, respectively. Statistical significance data not given. MMR: 0/1000 vs. 21.2/1000 live births in intervention and control groups, respectively. Statistical significance data not given.

Tumwine 1996 [107]	Zimbabwe. Rural district. Prospective cohort study. N = 1,053 pregnant women (N = 280 intervention group, N = 773 controls).	Compared the impact on pregnancy outcomes of women using a maternity waiting shelter (exposed group) vs. those coming directly to the hospital i.e. non-waiting mothers (unexposed).	PMR: 25.0/1000 vs. 29.8/1000 in intervention and control groups, respectively; P > 0.05 **[NS]**.

van Lonkhuijzen et al. 2003 [105]	Zambia. Rural setting. Prospective cohort study. N = 510 pregnant women (N = 218 exposed group, N = 292 non-exposed).	Compared the impact on risk status and pregnancy outcome in women staying at maternity waiting homes (exposed) with those women who gave birth in hospital after direct admission (unexposed).	PMR/MMR/Birth weight: No significant differences between the two groups. Spontaneous vaginal vertex delivery: 86% vs. 95% in the exposed and non-exposed groups, respectively.

Chandramohan et al. [106] evaluated the effect of a maternity waiting home on perinatal mortality in a large cohort of women (N = 6438) delivering at a district hospital in Zimbabwe. Waiting home users had a trend toward lower risk of perinatal death compared to direct hospital admissions (adjusted RR = 0.67; 95% CI: 0.40–1.05 [NS], P = 0.07). In the sub-group of women with antenatal risk factors (as assessed at hospital admission), there was a statistically significant 48% reduction in risk of perinatal death for waiting home users compared to women who traveled directly from home to the hospital during labour (adjusted RR = 0.52; 95% CI: 0.29–0.91; P < 0.05) ***[LOE 2++]***. A similar study conducted in rural Zimbabwe by Tumwine [107] reported that maternity waiting home use was associated with a non-significant reduction in PMR among waiting home users compared to direct hospital admissions (25/1000 vs. 29.8/1000, respectively, P > 0.05) ***[LOE: 2-]***. In Ethiopia, a study by Poovan et al [108](N = 777 women) found that women with high-risk pregnancies or who lived in remote areas who stayed at waiting homes had a stillbirth rate of 28.2/1000 (4/142 pregnancies ended in stillbirth) compared to 253.5/1000 (161/635 pregnancies ended in stillbirth) for controls admitted directly to hospital ***[LOE: 2-]***.

In India, Guruvare et al. [109] reported that the perinatal mortality rate in the catchment area encompassed by six satellite maternity waiting homes attached to a tertiary care hospital was 21/1000 live births after programme implementation compared to the national average of 70/1000 births ***[LOE: 3]***.

##### Conclusion

Few studies have tested the impact of maternity waiting home use on perinatal outcomes (Grade C evidence). The three studies considered here, which all included rural women either known or presumed to be at high risk of complications, suggest that maternity waiting home use may improve pregnancy outcomes for high risk women. Because none of the studies controlled for differences between waiting home users and those admitted directly to hospital, conclusions that can be drawn from these data are limited. The use of mothers' waiting homes is common in many Southern African countries but does not appear to have achieved high coverage elsewhere. Overall, this intervention is promising as a strategy to increase facility-based births, especially among the very poor and women with identified risk factors, and warrants further evaluation in large scale studies with more rigorous study designs.

#### Home birth with skilled attendance versus hospital birth for low-risk pregnancy

##### Background

In high- and moderate-income countries, most women deliver in hospital labour wards. When home births occur in high-resource settings, they are often deliberately planned by women who have low-risk pregnancies and the financial means and access to have a facility-based birth. Home births are primarily attended by midwives with a philosophical orientation toward birth as a normal physiological process. Key reasons for the decision to have a home birth include a preference for "natural" childbirth, a desire for minimal intervention, and preference for a familiar setting [110]. Supporters of home birth point to the numerous uncertainties about benefits and safety of many routine medical interventions. The potential lack of medical interventions available in the home in case of life-threatening complications has rendered planned home births controversial in many high-resource countries [111].

The constellation of factors leading women to choose planned home birth in low-and middle-income country settings differs from high-income country settings, and is largely a function of barriers to care including cost and distance; concerns about privacy, respect, and quality of care in facilities; as well as cultural preference for relatives or TBAs to assist with the birth. Maternal preferences as well as the safety of home-based birth may vary from setting to setting. Particularly in low-/middle-income countries, home-based births frequently occur in the presence of a family member or a TBA rather than a skilled birth attendant, which may limit or delay recognition of complications. Home births without skilled birth attendance or rapid access to emergency obstetric care in low-/middle-income countries are a well-known risk factor for adverse perinatal outcomes [112].

##### Literature-based evidence

The literature search identified two systematic reviews and 4 other intervention/observational studies (Table 9), all from high-income countries. Olsen et al [113] conducted a pooled analysis of controlled observational studies (6 trials, N = 24,092 participants) of selected and largely low-risk pregnant women delivering at home versus in facilities (Additional file 5). Perinatal mortality was comparable in the two groups (OR = 0.87, 95% CI: 0.54–1.41), and the home birth group had a lower frequency of low Apgar scores (OR = 0.55; 0.41–0.74) ***[LOE: 1++]***. More recently, a Cochrane review by Hodnett et al. [111] evaluated all RCTs or quasi-RCTs that compared the effects of a "home-like" institutional birth environment to conventional hospital care (6 trials, N = 8677 women) (Additional file 6). Maternal morbidities were all lower in the "home-like" group, including risk of vaginal/perineal tears (RR = 0.93, 95% CI: 0.88–0.97; 4 trials; N = 8415) and episiotomy (RR = 0.85, 95% CI: 0.74–0.99; 5 trials; N = 8529). A trend towards increased perinatal mortality in the home-like setting was identified (RR = 1.83, 95% CI: 0.99–3.38; 5 trials; N = 8529) but this interpretation of the limited data has been questioned [114]***[LOE: 1+]***.

**Table 9:** Impact of home births with skilled attendance versus hospital births on stillbirths and perinatal mortality

**Source**	**Location and Type of Study**	**Intervention**	**Stillbirths/Perinatal Outcomes**
***Reviews and meta-analyses***			

Hodnett et al. 2005 [111]	Australia, Scotland, UK, Sweden and Canada. Meta-analysis (Cochrane). 5 RCTs included (N = 8529 participants).	To assess the effects of care in a home-like birth environment (intervention) vs. care in a conventional labour ward (controls).	PMR: RR = 1.83 (95% CI: 0.99–3.38) **[NS]**. [41/5288 vs. 13/3241 in intervention and control groups, respectively].

Olsen 1997 [113]	Switzerland, USA, Essex, Australia. Meta-analysis (non-Cochrane). 6 controlled observational studies included (N = 24,092 low-risk pregnant women).	Assessed the safety of planned home birth backed up by a modern hospital system (study group) compared with planned hospital birth (controls).	PMR: OR = 0.87 (95% CI: 0.54–1.41) **[NS]**.

***Intervention studies***			

Janssen et al. 2003 [115]	Canada. University of British Columbia (Home Birth Demonstration Project). Intervention study. N = 2,178 pregnant women (N = 864 Home Birth Project clients, N = 571 midwife-attended hospital, N = 743 physician-attended hospital deliveries).	Compared the effect on the outcomes of women remaining eligible for home birth at the onset of labour (intervention) vs. those women meeting eligibility requirements for home birth but planning instead to deliver in hospital with either a midwife or physician in attendance (controls).	PMR: adj. OR = 2.50 (95% CI: 0.27–24.5) **[NS]** [0.3% vs. 0.1% in intervention vs. physician-attended hospital birth, respectively]. PMR: 0.3% vs. 0% in home birth vs. midwife-attended hospital birth respectively.

***Observational studies***			

Janssen 2002 [165]	Canada. Prospective cohort study. N = 2,176 pregnant women (N = 862 home birth, N = 571 midwife-attended hospital, N = 743 physician-attended hospital births.	Compared the impact on pregnancy outcomes of planned home births (exposed group) vs. planned hospital births either attended by midwife (unexposed # 1) or the physician (unexposed # 2).	SBR: 2 vs. 0 vs. 1 in the exposed, unexposed # 1 and unexposed # 2 groups, respectively. PMR: 3 cases in the home birth group (2 stillbirths and one neonatal death). PMR: RR = 2.5 (95% CI: 0.27–24.5) in exposed vs. unexposed # 2.

Tracy et al. 2007 [116]	Australia. Population-based study. Retrospective cohort. Women (N = 1,001,249) who gave birth in Australia during 1999 to 2002. Of these women, 21,800 (2.18%) gave birth in a birth center.	Compared the impact on perinatal mortality of giving birth in "alongside hospital" birth centers (exposed group) vs. birth in the hospitals (unexposed group).	PMR: 1.51/1000 vs. 10.03/1000 in exposed vs. unexposed groups, respectively (statistically significant). PMR: 1.4/1000 vs. 1.9/1000 among term births to primiparas in exposed vs. unexposed groups, respectively. PMR: 0.6/1000 vs. 1.6/1000 among term births to multiparas in birth centers vs. hospitals, respectively.

Wiegers et al. 1996 [117]	Netherlands. Prospective study. Women (N = 1836) and midwives (N = 97).	Compared the impact of planned home birth (study group) vs. planned hospital birth (controls).	PMR: 0/471 (0%) vs. 2/369 (0.5%) among primiparous women in the study vs. control groups, respectively. PMR: 4/669 (0.6%) vs. 0/327 (0%) among multiparae in the study vs. control groups, respectively. Multiparae had significantly better perinatal outcome for planned homebirths than planned hospital births (t = 4.75, p < 0.001).

In Canada, the Home Birth Demonstration Project was begun after professional midwives became nationally regulated and home birth became available in 1998. Reporting on this project, Janssen et al. [115] compared the outcomes of home births attended by professional midwives to outcomes of births to women eligible for home delivery but who planned hospital delivery. Rates of Caesarean section were significantly lower in the home birth group compared with physician-attended hospital births (adjusted OR = 0.3, 95% CI: 0.22–0.43), but prevalence of perinatal mortality and meconium aspiration syndrome were too low to give meaningful point estimates of risk ***[LOE: 2-]***.

In Australia, Tracy et al. [116] assessed perinatal mortality in "alongside hospital" birth centers, reporting significantly lower perinatal mortality associated with birth center births as opposed to hospital births (1.51/1,000 vs. 10.03/1,000) ***[LOE: 2+]***. In the Netherlands, where planned home birth is common, Wiegers et al. [117] investigated the association between the intended place of birth (home or hospital) and perinatal outcome in women with low-risk pregnancies after controlling for parity and social, medical, and obstetric background. In multiparous women, perinatal outcome was significantly better for planned home births than for planned hospital births (t = 4.75, P < 0.001) ***[LOE: 2+]***.

##### Conclusion

The Cochrane review comparing home-like versus conventional institutional settings for birth does not show any increased risk of perinatal mortality among planned home births with skilled care compared to hospital-based births (Grade C evidence), suggesting that for low-risk pregnancies, home birth with skilled care is a safe alternative to facility-based birth, and potentially leads to fewer unnecessary interventions. The available evidence is exclusively from high-income countries, however, and transferability of the findings to low-/middle-income country settings where home births without skilled attendants are common and ANC coverage is poor may not be appropriate, as caregivers have few opportunities to effectively triage high-risk women. Presence of a skilled birth attendant at home births could be a practical option to improve obstetric health care access, safety, and accessibility, particularly in areas without ready access to facilities, and for many women with uncomplicated deliveries, would potentially improve perinatal health outcomes. Studies that test the feasibility and impact on stillbirths/perinatal mortality of home-based births with skilled attendants are needed. Still, without a well-functioning health system including rapid emergency transport and access to operative delivery and blood transfusion, complications arising at home in the absence of a supportive environment would increase the risk of poor perinatal outcomes.

#### Perinatal audit

##### Background

Audit and feedback, the process of retrospectively assessing clinical performance (particularly in instances of poor health outcomes) and furnishing this information to clinicians, can be effective in improving professional practice. As the perinatal mortality rate is used as a crude indicator of quality of intrapartum and early postnatal care, perinatal audit can compare clinical practice against a defined standard of care, and subsequently recommend, implement and monitor changes to remedy deficiencies [118]. Perinatal audit systems may be conducted as hospital-based case review using any of a range of methods and classification criteria to change provider practices, or may involve confidential national enquiries using population-based regional or national data to formulate guidelines and improve standards of care [119]. Most confidential enquiries are conducted on a regional basis, but the UK has an exemplary national perinatal audit system called the Confidential Enquiry into Maternal and Child Health (CEMACH) [120,121]. South Africa is the only low-/middle income country with a confidential enquiry for maternal deaths, and also a voluntary perinatal audit system which now covers 40% of the births nationally. This provides invaluable data on avoidable causes of death – at the provider interface, administratively, and in the community [122].

##### Literature-based evidence

We identified two systematic reviews and 13 other intervention/observational studies (Table 10). Pattinson et al. [123] proposed a meta-analysis of RCTs of audit and feedback that reported objectively measured professional practice in a health care setting or health care outcomes. Unfortunately, no studies met the selection criteria. A previous systematic review by Mancey-Jones et al. [124] (Additional file 7) described how the impact of audit on perinatal outcome in low-/middle-income countries has usually been assessed by before-and-after time series analyses, with some studies reporting statistically significant improvements in crude perinatal mortality rates after the introduction of regular audit [125-128]. High proportions of intrapartum fetal deaths were reported to be associated with avoidable factors [126-129], and showed a significant reduction in some studies after audit was introduced [126,130]. All reports concluded that the perinatal audit process contributed to improved perinatal care ***[LOE 1+]***.

**Table 10:** Impact of perinatal audit on stillbirths and perinatal mortality

**Source**	**Location and Type of Study**	**Intervention**	**Stillbirth/Perinatal Outcomes**
***Reviews and meta-analyses***			

Mancey-Jones and Brugha 1997[124]	Zimbabwe, Guadeloupe, South Africa, Mozambique. Systematic review. Before-and-after time series analyses. 7 studies included from low-/middle income countries.	Assessed the impact on perinatal mortality rates before and during a perinatal audit.	No pooled analysis done. PMR (Guadeloupe): fell by 25% during audit. PMR (Lebowa, South Africa): significant reduction. PMR (Mozambique): no change (increase in high risk patients). PMR (Port Elizabeth, South Africa): significant reduction.

***Intervention studies***			

Biswas et al. 1995 [166]	Singapore. National University Hospital. Comparison of perinatal mortality rates between two time periods. N = 26,173 mothers with N = 26,423 births during 1986–1992. N = 235 perinatal deaths, of which 145 (61.7%) were stillbirths and 90 (38.3%) were neonatal deaths.	Compared the perinatal mortality rate during a 7-year period (1986–1992) vs. baseline assessed in 1982 using perinatal audit. Compared to 1982, 1986–1992 were marked by improvements in antenatal and intrapartum fetal surveillance and improved neonatal care.	PMR: 8.9/1000 vs. 14.6/1000 in during perinatal audit period vs. baseline. PMR (excluding lethal malformations): 5.7/1000 vs. 14.6/1000 in 1986–1992 vs. 1982

Cameron et al. 2001 [167]	Australia (Far North Queensland). Atherton Hospital. Before-and-after study design. N = 5,879 births (N = 2996 during 1991–00, N = 2883 during 1981–90).	Analysed obstetric audit data collected from 1991–2000, comparing PMR during this period with the previous decade (1981–90). Was associated with increased public sector utilization and caesarean section rate (13% to 17.4%)	SBR: 12/2996 (4/1000) vs. 7/2883 (2.4/1000) after vs. before, respectively. PMR: 16 (5.3/1000) vs. 15 (5.2/1000) after vs. before, respectively. NMR: 4/2996 (1.3/1000 live births) vs. 8/2883 (2.8/1000) after vs. before, respectively. No statistical significance data given.

Cameron 1998[168]	Australia (Far North Queensland). Atherton Hospital. Descriptive study. N = 2883 deliveries during 1981–1990 (N = 1974 public confinements, N = 909 private confinements).	Analysed obstetric audit data collected from private vs. public facilities over the decade 1981–1990.	PMR: 5.2/1000. PMR: 5.1/1000 vs. 5.5/1000 in public and private confinements, respectively. PMR (corrected): 9.6/1000 vs. 13.5/1000 vs. 16.9/1000 in public patients, Queensland (1987) and the Far North Statistical Division (1987).

Dahl et al. 2000 [169]	Norway (Troms County). Medical Birth Registry of Norway and medical records. Retrospective + prospective study design. N = 472 antenatal, neonatal and post neonatal deaths = 20 weeks of gestation from 1976– 1997.	Evaluation of deaths, including assessment of risk factors, mortality rates, cause of death, sub-optimal care and avoidable deaths, by medical audit to improve antenatal and neonatal care over a 22-year period.	Fetal death (miscarriage + SB) + NMR + IMR: 13.8/1000 in 1976–80 9.5/1000 in 1981–85 10.4/1000 in 1986–91 7.7/1000 in 1992–97 (P < 0.001) Reduction attributable to reduced rate of pre-term birth (P < 0.001) and low birth weight (500–1995 g) (P < 0.001).

Hawthorne et al. 1997 [170]	United Kingdom. District general and teaching hospitals (Population data). Prospective audit. N = 111 diabetic pregnant women booking in 1994.	To compare perinatal mortality associated with diabetic pregnancies with the background population (controls) to determine progress toward a specified target of diabetic pregnancy outcome approximating non-diabetic pregnancy outcome.	PMR: OR = 5.38 (95% CI: 2.27–12.70). [48/1000 vs. 8.9/1000 in diabetic pregnancy and controls, respectively]. NMR: OR = 15.0 (95% CI: 6.77–33.10). [59/1000 vs. 3.9/1000 in the diabetic pregnancies vs. controls, respectively].

Jansone and Lazdane 2006 [171]	Latvia (Riga). Tertiary referral perinatal care center. Retrospective audit. N = 26,783 births, of which N = 494 were stillbirths and neonatal deaths during 1995–1999.	To analyze all perinatal deaths using the Nordic-Baltic classification system, and assess trends in perinatal mortality over the study period.	PMR: No decline during the study period. Proportion of preventable perinatal deaths: 36.4% vs.14.7% in 1999 vs. 1995, respectively (P = 0.01).

Korejo et al. 2007 [172]	Pakistan (Karachi). Government teaching hospital. Prospective review. N = 7743 deliveries in 2001, of which N = 753 were perinatal deaths (N = 569 stillbirths and N = 184 early neonatal deaths).	To review the extent and determinants of perinatal mortality using the Aberdeen classification system, which analyses cause of death as well as preventive factors, comparing the PMR in 2001 with previous data.	SBR: 73.4/1000 total births. PMR: 97.2/1000 total births. No change in PMR over 40 years due to higher patient influx and incoming referrals. Perinatal deaths associated with poor care and education and low socio-economic status.

Krebs et al. 2002 [136]	Denmark (Copenhagen). University hospital. Blinded controlled perinatal audit). N = 12 non-malformed, breech infants with intrapartum/early neonatal death in the period 1982–92. N = 23 controls matched by presentation and planned mode of delivery.	11 obstetricians reviewed the data (derived from maternity records) and narratives of cases and controls, subsequently completing questionnaires in which they guessed whether the baby had died based on the data (for both cases and controls), and whether suboptimal care had been provided during pregnancy and delivery, indicating a potentially avoidable death.	Suboptimal ANC: 17% vs. 4% in cases and controls, respectively. Suboptimal intrapartum care: 25% vs. 26% in cases and controls, respectively. When death was assumed, obstetricians asserted it was potentially avoidable in 7/12 (58%) of cases and 4/23 (17%) of controls (P = 0.02).

Krue et al. 1999 [173]	Denmark (Viborg County). Perinatal audit (county-wide).	Compared perinatal mortality over a three-year perinatal audit period from 1994–1996. The mortality rate in 1995 was also compared to data from the Danish National Birth Register (1995).	PMR: 6.5/1000 vs. 9.4/1000 in 1996 vs. 1994, respectively **[NS]**. NMR: 2.4/1000 vs. 3.2/1000 in 1996 vs. 1994, respectively **[NS]**. PMR: No difference between county and national rates in 1995.

Papiernik et al. 2005 [174]	France (Paris). Perinatal audit. All deaths from 1989 to 1992 in the Perinatal Enquiry.	Assessed the impact of audit of obstetrical practices and the analysis of perinatal deaths on perinatal mortality.	PMR: Major reduction after a 10-year period.

Tay et al. 1992 [137]	Singapore. Tertiary referral hospital. Caesarean audit. N = 16, 875 deliveries during the 4 year period.	Assessed the impact on perinatal mortality of an intradepartmental audit (critical review of indications for cesarean delivery).	PMR: 8.25, 7.05, 9.39 and 5.38 in 1987, 1988, 1989 and 1990, respectively for infants weighing ≥ 500 g Caesarean section rate: 12.3%, 11.1%, 11.2% and 11.4% for 1987, 1988, 1989 and 1990, respectively.

Wilkinson et al. 1997 [135]	South Africa (rural). Hlabisa Maternity Service, comprising Hlabisa Hospital, 8 village clinics, and 20 mobile clinic points. Perinatal audit with subsequent interventions. N = 21,112 consecutive births between May 1991 and December 1995.	To assess the impact of the perinatal audit on the quality of care, along with the design of interventions informed by the audit results. The interventions employed consisted of structural and functional rearrangement of the maternity service district-wide, writing and implementing protocols of care for local use, and regular in-service education.	PMR: 27/1000, 42/1000 and 26/1000 in 1991, 1992 and 1995, respectively (40% reduction from the peak in 1992; P = 0.002). PMR: 653/21,112 (31/1000) from 1991 to 1995. Proportion of perinatal deaths occurring in clinics (vs. at home/outside of clinics): 6.3% vs. 17% in 1995 vs. 1991, respectively.

**Observational studies**			

King et al. 2006 [120]	Australia (Victoria). Maternity hospitals. Perinatal death audit. A cohort of N = 3485 perinatal deaths over a 5-year period, 2000–2004. Live births: N = 312651; stillbirths: N = 242; neonatal deaths: N = 1057	To assess the impact of a systematic audit of stillbirths and neonatal deaths via application of the classification systems developed by the Perinatal Society of Australia and New Zealand (PSANZ).	Causes of perinatal deaths: congenital abnormality (24.5%), followed by spontaneous preterm birth (17.0%), unexplained antepartum death (15.9%), and maternal conditions (14.8%). Causes of stillbirths: unexplained antepartum death was the main cause of death (22.9%), followed by congenital abnormality (20.3%) and maternal conditions (20.4%).

Several other observational studies support the value of clinical audit in improving health practice and/or perinatal outcomes [119,120,124,131-134]. In rural South Africa, Wilkinson et al. [135] assessed the impact of perinatal audit using data from 21,112 consecutive births from 1991–1995. Although the average number of deliveries increased by 31%, perinatal deaths with birth weight of at least 1000 g declined steadily over the project period from a peak in 1992 of 42/1000 to 26/1000 in 1995 (40% reduction; P = 0.002). The proportion of avoidable deaths fell from 19% in 1991 to zero in the second half of 1995 (P = 0.0008) ***[LOE: 2+]***. In Denmark, Krebs et al. [136] conducted a blinded controlled audit with 11 obstetricians of all cases of intrapartum stillbirth or early neonatal death of normally formed term breech births from 1982–1992, in which a narrative of care was read for each delivery and the obstetricians were asked to guess whether the infant had died, and whether the death was avoidable. The majority guessed that 42% of cases and 9% of the controls had died, and concluded that the death was potentially avoidable in 58% of cases versus only 17% of controls ***[LOE: 1-]***. In Singapore, Tay et al. [137] conducted a Caesarean section audit to reduce rates of unnecessary Caesarean sections based on clinical indications, and found that the overall caesarean section rates and perinatal mortality rates were relatively steady from 1987–1990, but caesarean section for cephalo-pelvic disproportion decreased by 26.8% (P = 0.0013) from 1987 to 1990 ***[LOE: 3]***.

##### Conclusion

Although in other applications, audit and feedback have not been consistently found to be effective [138], several RCTs of perinatal audit processes and feedback reported significant improvements in professional practice and/or reductions in perinatal mortality [125-128,130,139]. Our review indicates that perinatal audit can effectively identify problems in overall obstetric care, and implementation of subsequent changes in practice is often followed by measurable declines in perinatal mortality (Grade B evidence). There are relatively few large-scale studies of perinatal audit from district health systems in low-/middle-income countries; conducting such studies might highlight the preventability of stillbirths and mobilise community demand for quality improvement. Perinatal audit is a helpful diagnostic strategy on which to base quality improvement initiatives, and can be readily integrated with maternal audit systems [140].

## Discussion

### Summary of evidence to improve service supply and community demand for interventions to prevent stillbirth

Several innovative strategies have been examined to promote the utilization and quality of interventions to improve perinatal outcomes and prevent stillbirth; however, few have been tested at scale. These strategies include community-based schemes to generate demand and finance care where cost is a barrier, efforts to upgrade or evaluate the skills of health care providers, and innovative ways of re-organizing care to make obstetric care – particularly the subset of interventions that constitute emergency obstetric care – more accessible, affordable, and effective. The evidence for health systems strategies to improve uptake and quality of interventions to prevent stillbirth is summarised in Table 11.

**Table 11:** Collective grading of evidence for impact of health systems and human resource interventions on stillbirth and related perinatal outcomes

	**Evidence of no or negative impact** (leave out of programs)	**Uncertain evidence** (need for additional research before including in programs)	**Some evidence** (may include in programs, but further evaluation is warranted)	**Clear evidence** (merits inclusion in programs)
Emergency loan/insurance funds for obstetric emergencies		X		

Financial incentives to improve access to emergency obstetric care				X

Training traditional birth attendants in clean delivery and referral				X

Training of other cadres of community health workers			X	

Training nurse-aides as birth attendants (including Caesarean section)		X		

Training professional midwives in antenatal and intrapartum care			X	

Obstetric drills		X		

Training in neonatal resuscitation for physicians and other health workers			X	

Public-private partnerships		X		

Maternity waiting homes		X		

Home birth with skilled attendance versus hospital birth			X (high-income country studies only; no disadvantage to home birth)	

Perinatal audit			X	

Community demand-side interventions to improve accessibility and uptake of facility-based care appear to be effective in many settings, particularly where quality facility-based care is available but cost of services or transport impedes care-seeking. Although most studies of community-based loan/insurance schemes and financial incentives involve populations which are too small to assess statistically significant changes in birth outcomes, they offer promising models of improving accessibility of care that may improve care-seeking while sparing families from catastrophic household expenditures. Similarly, maternity waiting homes have not yet shown a demonstrable impact on stillbirths due to limitations in size and design of the studies on this subject. The reported maternal and infant benefits of maternity waiting homes suggest that their availability for primiparas and high-risk pregnancies in areas with poor access to emergency obstetric care might prevent stillbirths. Sustainability of these schemes is challenging; public-private partnerships may offer one possible option for financing care.

Although evidence from rigorous studies is limited, a number of studies suggest that cadres of health workers other than physicians have an important role to play in the prevention of stillbirths. The impact of training TBAs in clean delivery and management of birth asphyxia is small but significant, suggesting that this important human resource should not be overlooked in community-based efforts to improve birth outcomes. TBAs can also aid in the transfer of women with complications to health facilities, particularly in settings where TBAs are widely utilised and where the formal health system is dysfunctional or nonexistent. In certain settings where doctors are unavailable, task-shifting to other cadres of health workers to perform Caesarean section and neonatal resuscitation may be feasible, low-cost and effective [71,73,72,74]. However, limited data are currently available to indicate mortality impact for maternal, stillbirth and neonatal outcomes and further research is needed, especially outside Africa.

Quality of facility-based care is critical to prevent stillbirths, particularly intrapartum stillbirths. Perinatal audit offers an important tool to systematically review stillbirth cases and to improve quality by changing provider behavior, hospital policy, and/or national guidelines, and has shown evidence of impact in a variety of settings [141].

In low-/middle-income countries, the demonstration projects with the largest impact on stillbirth and perinatal mortality have been those that galvanise stakeholders from communities, health systems, government, donors, and/or the policy community and implement a package of biomedical interventions, coupled with community mobilisation and health systems strengthening. The MotherCare demonstration projects in Bolivia, Guatemala, Indonesia, and Nigeria [49] and the large declines in stillbirths in peri-urban slums in Pakistan by Jokhio et al. [47] offer illustrative examples of how community activism, improved provider skills, and efforts to overcome infrastructural limitations can act synergistically to reduce perinatal mortality.

Delivery approaches must be tailored to setting-specific needs and resource constraints. For example, in secondary or tertiary-care facilities in middle- or high-income countries, skills training in neonatal resuscitation for physicians in conjunction with perinatal audit may be sufficient to bring about significant improvements in quality of care for asphyxiated infants who would otherwise be misreported as stillbirths. In low-/middle-income-country settings where home births are common, facility-based care is perceived to be of poor quality, and financial barriers to accessing care are high, community-based demand creation strategies such as loan schemes and health promotion using community health workers could complement interventions to improve quality of care through increasing capabilities of providers, health systems strengthening, and/or perinatal audit. Where improving access to facilities for intrapartum care is geographically infeasible, it may be possible to upgrade the skills of TBAs to conduct clean delivery while working to link them with the formal health system and to raise community awareness of the preventability of perinatal death and the importance of ANC and birth preparedness. Whether skilled birth attendants can effectively triage and refer high-risk births in rural areas of low-/middle-income countries, while safely attending low-risk births at home or in birthing centres, has not been adequately tested. Current evidence suggests that in high-income countries, perinatal outcomes are similar between home and hospital settings for low-risk pregnancies. In instances where access to emergency obstetric care is limited, attempting intervention approaches such as emergency transport or maternity waiting homes, while expanding access to emergency obstetric care through innovative public-private sector linkages, can potentially improve uptake of care and provide a safety net for women who deliver at home or in birthing centres without emergency obstetric services.

### Delivering effective interventions to prevent stillbirths: summary of recommendations for programmes (Table 12)

**Table 12:** Key programme recommendations to reduce stillbirths

1. Community demand creation strategies and training of appropriate human resources for health promotion and preventive interventions.
2. Antenatal care to deliver quality interventions and to screen for high risk pregnancies
3. Recognition and management of maternal infections during pregnancy, such as syphilis and malaria
4. Skilled attendance at birth and emergency obstetric care availability (including Caesarean section)

The previous five papers in this series have evaluated a range of behavioural, nutritional, clinical, and monitoring interventions across the continuum of care from pre-conception through the intrapartum period. A summary of the evidence base for the interventions we reviewed in the first five papers has been presented in Figure 1. Only 5 interventions showed clear evidence of impact on stillbirth incidence. There was some evidence for an impact of 9 other interventions, but a lack of study rigor and/or insufficient numbers of studies suggest that further evidence is needed before their impact on stillbirths can be assessed conclusively. We found either a lack of evidence or no evidence of impact for the remaining 46 interventions we considered, revealing the lacunae in our understanding of which interventions can reduce stillbirth, and by what measure. Many studies were underpowered to detect impact on stillbirths, having been designed to measure other outcomes. In many instances we were forced to rely exclusively on observational studies, although in some instances current practice guidelines strongly support an impact on stillbirths: for example, provision of emergency obstetric care and intrapartum monitoring with access to Caesarean section.

Table 13 details the potential care providers and delivery strategies for promoting various interventions with some or clear benefit of impact on stillbirths or perinatal mortality, and which we short-listed for scaling up or further evaluation. Some of these, such as anti-helminthic treatment or malaria chemoprophylaxis, are deliverable as part of available routine packages of care through outreach or routine ANC, whereas others, such as insecticide-treated bed net use, might also require special promotional or behaviour change strategies such as mass media campaigns at the community level.

**Table 13:** Possible delivery strategies for interventions with some or clear evidence of impact on stillbirths

	**Mass media (including social marketing strategies, health days, etc)**	**Facilitated community and advocacy groups**	**TBAs**	**Trained CHWs (outreach workers)**	**Community-based professional midwives**	**Other cadres of facility-based health workers**	**Medical/nursing staff in first-level facilities**
Multiple micronutrient supplementation		+	+	✓	✓	✓	✓

Balanced protein-energy supplementation		+	+	✓	✓	✓	✓

Anti-malarials in pregnancy	+	+		✓	✓	✓	✓

Insecticide-treated bed nets in pregnancy	+	✓	✓	✓	✓	✓	

Heparin in pregnancy for clotting disorders and antiphospholipid syndrome							✓

Anti-helminthic treatment in pregnancy				✓	✓	✓	✓

Syphilis screening and treatment in pregnancy				+	✓		✓

Management of intrahepatic cholestasis in pregnancy							✓

Fetal movement counting (in high-risk pregnancy)			+	+	✓	✓	✓

Doppler monitoring (in high-risk pregnancy)							✓

Intrapartum cardiotocography (with access to Caesarean section)							✓

Amniotic fluid volume assessment in pregnancy							✓

Emergency obstetric care packages, including Caesarean section		+	+	+	+	✓	✓

Elective induction of labour in post-term pregnancies			+		+		✓

Planned Caesarean section for term breech presentation*					+		✓

Perinatal audit		✓			✓		✓

✓ Provision of intervention

+ Promotion of intervention

* Only advised in areas with ability to perform safe caesarean section

We propose some clear priorities for promoting the delivery of interventions in programmes. Firstly improving coverage of skilled birth attendance and emergency obstetric care is the top priority, as skilled attendance is consistently associated with reductions in intrapartum stillbirth and decreased maternal mortality [142]. However, there are some areas of the world with such a dearth of skilled birth attendants that even aggressive and developed training programmes cannot achieve a high proportion of births with skilled attendance in the short term [143,144]. In these situations, there is positive but limited evidence from this review that training different cadres of community workers, including TBAs, to provide basic clean childbirth care and to refer complications has the potential to have a small positive impact on birth outcomes. TBAs have been successfully trained in some studies to work in tandem with other health workers and can build effective linkages with health systems for referrals [56,60].

Disappointingly, few interventions to detect problems late in pregnancy and during labour are supported by rigorous evidence from RCTs. In general, the effectiveness of screening procedures and monitoring techniques is limited, particularly for low-risk pregnancies, and none of the interventions reviewed can be recommended presently for inclusion in programs. There is a need for further research to confirm whether identification and appropriate management of high-risk pregnancies, including multiple pregnancy, breech presentation, and pregnancies with evidence of inadequate fetal growth; and effective management of maternal conditions such as diabetes, hypertensive disorders, and clotting disorders, can effectively prevent stillbirths. Improving rates of skilled attendance at birth and access to safe, high-quality emergency obstetric care are of paramount importance, as women without access to such care have a well-documented increased risk of stillbirth [145-147]. Observational and historical data from high-income countries suggests that the introduction of fetal monitoring in conjunction with the availability of Caesarean section for fetal distress has led to significant declines in stillbirth rates [148-150], suggesting that monitoring technologies may be effective in preventing stillbirth where safe Caesarean section is or can be made available. Because Caesarean section carries higher risks of maternal morbidity and mortality, as well as adverse outcomes in subsequent pregnancies if access to care is poor; it is thus recommended that in low-resource settings, Caesarean section be conducted only when clinically indicated.

The second priority is to address maternal infections, especially syphilis and malaria in endemic areas. Maternal infections are estimated to contribute to 25–50% of stillbirths in low-/middle-income countries, although systematic estimates are lacking [151]. In areas where syphilis prevalence is high or malaria is endemic, interventions to prevent or treat these infections are a major weapon in the arsenal to reduce stillbirth incidence. In some areas of southern Africa, one-quarter to one-half of all stillbirths occur in women seropositive for syphilis, and it is estimated that in these settings, 25% of all stillbirths may be caused by syphilis [152]. Evidence from Tanzania suggests that effective syphilis treatment could dramatically reduce stillbirth rates where prevalence is high, as treatment reduced the risk of stillbirth to rates comparable to those observed among women without syphilis infection [153]. Programmes must grapple with logistical challenges to screening and treating syphilis infection, as well as partner identification.

Nearly 40% of the world's population lives in malaria-endemic areas, a known risk factor for stillbirth [154]. Intermittent preventive treatment of malaria and use of insecticide-treated bed nets during pregnancy can protect women from maternal malaria during pregnancy and reduce stillbirth rates among women in their first or second pregnancy; these interventions can be provided using a combination of outreach and community-based strategies.

Thirdly, quality ANC offers an entry point to the health system and may increase the likelihood that women obtain timely emergency obstetric care [142]. An ANC contact alone is of uncertain benefit for stillbirth reduction; further research is needed to identify the most effective components of antenatal care, and the extent to which this platform can be built upon to increase utilisation of skilled intrapartum care.

An important linked priority is to promote demand for services through a range of CHWs operating through outreach programmes and working in tandem with community groups [56,60]. Communities themselves have many resources available to improve perinatal outcomes; many interventions recommended for scaling up either can be performed by community workers with limited training, or would be rendered more effective alongside community-based efforts to improve uptake or accessibility at the community level. Continuing to emphasise, promote, and incentivise skilled birth attendance wherever feasible; early recognition of complications both antenatally and during labour, and community-facility strategies to overcome barriers to care and facilitate rapid referral to emergency obstetric care for complications could prevent many stillbirths in low-/middle-income countries.

### Delivering effective interventions to prevent stillbirths: recommendations for policy and research

Although stillbirth is one of the most common adverse outcomes of pregnancy, stillbirth has been largely overlooked by policy makers and researchers. The dearth of studies for most interventions we reviewed illustrates the lack of priority historically afforded to stillbirth research. There is convincing evidence from RCTs and observational studies that delivery at scale of interventions such as skilled attendance at birth, including intrapartum monitoring with access to operative delivery, and prevention and treatment of syphilis and malaria can bring about clinically significant reductions in stillbirth rates, but political will to invest in these interventions is still lacking.

One reason for this hampered progress toward reducing stillbirths is the research gaps that surround stillbirth causation and prevention. Currently the findings of the few studies reporting stillbirth as an outcome typically had other primary outcomes and were often underpowered to detect impact on stillbirths, or had poor generalisability to different health systems and circumstances. Donors and governments need to be encouraged to commit to funding large-scale efficacy and effectiveness studies of interventions that could prevent stillbirth. Investigators of large RCTs of interventions with potential impact on stillbirths should be encouraged to collect data on and report stillbirths with their study findings, disaggregated from the vague composite measure of perinatal mortality. Some risk factors such as malnutrition are well-documented, but strategies to improve maternal nutritional status and thereby reduce stillbirth incidence have met with limited success. Other risk factors and causes of stillbirths are still unknown, and there is insufficient evidence to recommend a number of potentially promising interventions to treat risk factors for stillbirth, including diabetes, maternal hypertensive disorders, and chorioamnionitis. Reducing chorioamnionitis is of particular import, as more than 50% of early stillbirths in all settings are associated with chorioamnionitis.

Many stillbirths could be prevented if pregnant women had access to quality peri-conceptional care and ANC during pregnancy, skilled attendance at birth, and emergency obstetric care for complications. Committing funding to expand coverage and quality of these services is imperative. The implementation of such improvements is bedeviled by logistical challenges in identifying risk, securing rapid transport, and providing timely intervention at facilities [147]. In areas where provision of such care is presently difficult or impossible, there is a need to develop innovative ways of "bridging the gaps" to bring quality antenatal and intrapartum care to poor rural and urban women. Financial schemes to reduce financial barriers and maternity waiting homes and emergency transport schemes to reduce physical access barriers are promising approaches that merit further testing. Locally tailored versions of emergency obstetric drills for shoulder dystocia and Caesarean section may prove an effective performance improvement strategy even in fairly resource-poor settings; however, this strategy has not yet been tested.

In strategically advocating for stillbirth prevention, existing maternal and newborn health initiatives can and must be galvanised to include stillbirth prevention as part of their advocacy for resources. Many of the interventions that can impact stillbirths are also of benefit to mothers and newborns, including access to emergency obstetric care, screening and treatment of syphilis and measures to reduce the burden of malaria (Table 14). Improved maternal nutrition during pregnancy positively impacts maternal health status, and may affect long-term developmental outcomes of the child after birth. As an illustrative example, although there is limited evidence that balanced protein-energy supplements can reduce stillbirth rates [155], the evidence base indicates that these supplements reduce rates of low birth weight, which may improve long-term outcomes significantly. Micronutrient supplementation containing iron can reduce rates of maternal anaemia and correct other micronutrient deficiencies; and although its impact on stillbirths remains inconclusive, periconceptual folic acid intake can significantly reduce rates of neural tube defects in the newborn [156], thereby potentially reducing infant morbidity and mortality [157]. Advocacy initiatives can draw on this complementarity of maternal and newborn health with stillbirth interventions to strengthen arguments for increases in global funding for antenatal and intrapartum services.

**Table 14:** Interventions with evidence of synergistic impact on stillbirth and maternal and neonatal health outcomes

	**Evidence of stillbirth reduction**	**Maternal benefit**	**Neonatal/infant benefit**
Antenatal multiple micronutrient supplementation	*	Reduced anemia (iron-containing supplements)	Improved micronutrient status and survival

Balanced protein-energy supplementation	*	Improved nutritional status	Reductions in low birth weight

Maternal deworming	*	Reduced anemia, improved nutritional status	

Syphilis screening and treatment	**	Eradication of syphilis infection	Reductions in congenital syphilis (neonatal morbidity and mortality)

Malaria chemoprophylaxis	*	Reduced burden of malaria and anemia, reduced maternal mortality	

Insecticide-treated bed net use in pregnancy	**	Reduced burden of malaria and anemia, reduced maternal mortality	

Intrapartum cardiotocography with or without pulse oximetry	*	-	Reduced birth asphyxia, neonatal mortality

Emergency obstetric care packages	**^1^	Reduced maternal morbidity and mortality	Reduced neonatal morbidity and mortality

* Some evidence of benefit

** Clear evidence of benefit

^1^Based on observational data

Under-reporting of stillbirths, a lack of data on population-attributable fractions of stillbirth by cause, and a lack of priority accorded to investigating the role of care providers in preventing perinatal deaths all contribute to a lack of global priority for stillbirth research and interventions. There is a need for better surveillance and ascertainment of stillbirth incidence at all levels of health facilities and in communities where stillbirth is a private and stigmatised event that is often hidden from view. Verbal autopsy strategies offer one potential means of identifying causes of stillbirths at community level. In facilities, particularly those with adequate diagnostic and laboratory capacity, standardised templates and classification schemes may help better document stillbirths and shed light on the population-attributable fractions of stillbirths associated with particular causes and risk factors. To improve quality of care in hospitals, districts, and regions with high rates of stillbirths and perinatal mortality, we recommend further research regarding the impact of emergency obstetric drills on provider practices and perinatal mortality outcomes, as well as more widespread use of perinatal audit systems. Perinatal audit systems hold considerable promise for improving maternal and newborn outcomes [87,124,139,158]. Audit recommendations can bring about improvements in case management, hospital policies, standards of care, and national guidelines that can help improve quality of care and consequently reduce the burden of preventable stillbirths and perinatal mortality.

### Conclusion

While large evidence gaps remain, there is a compelling case that scaling up several interventions, particularly emergency obstetric care packages; screening and treatment for maternal infections especially syphilis, and malaria prevention and treatment could substantially reduce the burden of stillbirths in low-/middle-income countries. We also have parallel evidence from a range of delivery strategies addressing barriers to access to care, and task-shifting, including use of alternative cadres of non-physician health workers, which suggests that these interventions can be implemented and scaled up in situations where they are most needed. Key data gaps also remain, particularly effective tracing of stillbirths in national household surveys and the establishment of a classification system that ensures visibility and available data for action. However, the largest remaining gap is for more widespread recognition and political commitment to reduce this massive loss of life of at least 3.2 million stillbirths, 1 million of which occur right at the time of birth.

## Abbreviations

ACOG: American College of Obstetricians and Gynaecologists; ANC: antenatal care; CEMACH: Confidential Enquiry into Maternal and Child Health; CHW: community health worker; JSY: Janani Suraksha Yojana; LOE: Level of Evidence; OR: odds ratio; RCT: randomised controlled trial; RR: relative risk; TBA: traditional birth attendant; WHO: World Health Organization.

## Competing interests

The authors declare that they have no competing interests.

## Authors' contributions

All authors contributed to the evidence review, conceptualization and writing of this paper.

## Supplementary Material

Additional file 1 **Web Table 1. Component studies in Sibley et al. 2007: Impact of trained birth attendants on stillbirth and perinatal mortality**. Component studies in Sibley et al. 2007 meta-analysis reporting impact on stillbirths/perinatal mortality. Click here for file

Additional file 2 **Web Table 2. Component studies in Hodnett and Fredericks 2003 meta-analysis: Impact of support during pregnancy by health workers and midwives on stillbirth/neonatal mortality**. Component studies in Hodnett **and Fredericks** 2003 meta-analysis reporting impact on stillbirths/perinatal mortality. Click here for file

Additional file 3 **Web Table 3. Component studies in Villar et al. 2001 meta-analysis: Impact of different patterns of antenatal care on perinatal mortality**. Component studies in Villar et al. 2001 meta-analysis reporting impact on stillbirths/perinatal mortality. Click here for file

Additional file 4 **Web Table 4. Component studies in Hodnett 2000 meta-analysis: Impact of continuous care by caregivers during pregnancy and childbirth on stillbirth/neonatal mortality**. Component studies in Hodnett 2000 meta-analysis reporting impact on stillbirths/perinatal mortality. Click here for file

Additional file 5 **Web Table 5. Component studies in Olsen et al. 1997 meta-analysis: Impact of planned home versus hospital births on perinatal mortality**. Component studies in Olsen et al. 1997 meta-analysis reporting impact on stillbirths/perinatal mortality. Click here for file

Additional file 6 **Web Table 6. Component studies in Hodnett et al. 2005 meta-analysis: Impact of home-like versus conventional settings for birth on perinatal mortality**. Component studies in Hodnett et al. 2005 meta-analysis reporting impact on stillbirths/perinatal mortality. Click here for file

Additional file 7 **Web Table 7. Component studies in Mancey-Jones and Brugha 1997: Impact of perinatal audit on perinatal mortality**. Component studies in Mancey-Jones and Brugha 1997 meta-analysis reporting impact on stillbirths/perinatal mortality. Click here for file
