# Supplementary material for: Delivering interventions to reduce the global burden of stillbirths: improving service supply and community demand
Source: BMC Pregnancy Childbirth. 2009 May 7;9(Suppl 1):S7. doi: 10.1186/1471-2393-9-S1-S7 (PMC2679413; doi:10.1186/1471-2393-9-S1-S7)
Supplement: Additional file 1 — Web Table 1. Component studies in Sibley et al. 2007: Impact of trained birth attendants on stillbirth and perinatal mortality. Component studies in Sibley et al. 2007 meta-analysis reporting impact on stillbirths/perinatal mortality. [file 1471-2393-9-S1-S7-S1.doc]

**Web Table 1. Component studies in Sibley et al. 2007 [1]: Impact of trained birth attendants on stillbirth and perinatal mortality**

| **Source** | **Location and Type of Study** | **Intervention** | **Stillbirths / Perinatal Outcomes** |
| --- | --- | --- | --- |
| 1. Jokhio et al. 2005 [2] | Pakistan. 7 rural subdistricts (talukas).  Cluster-RCT. N =19,557 pregnant women recruited. | To assess the impact on pregnancy outcome of intervention where the TBAs were trained and issued disposable delivery kits; Lady Health Workers linked TBAs with established services and documented processes and outcomes; and obstetrical teams provided outreach clinics for ANC. Women in the control areas received usual care. | SBR: adj. OR=0.69 (95% CI: 0.57-0.83); P<0.001.  [50/1000 (483/9710) vs. 71/1000 (638/8989) live births and stillbirths in intervention and control clusters, respectively].  PMR: adj. OR=0.70 (95% CI: 0.59-0.82); P<0.001.  [85/1000 (823/9710) vs. 120/1000 (1077/8989) live births and stillbirths in intervention and control groups, respectively].  NMR: adj. OR=0.71 (95% CI: 0.62-0.83); P<0.001.  [37/1000 (340/9710) vs. 53/1000 (439/8989) live births in intervention and control clusters, respectively].  MMR: adj. OR=0.74 (95% CI: 0.45-1.23); P=0.24 **[NS]**.  [268/100,000 (27/9710) vs. 360/100,000 (34/8989) pregnancies in intervention and control groups, respectively]. |
| 2. O’ Rourke 1994 [3] | Rural Guatemala.  Controlled before-after study. N=521 women who were referred to a health service. | Compared the impact of additional training of TBAs (intervention) vs. basic training (controls). | PMR: OR=1.02 (95% CI: 0.59-1.76); P=0.9 **[NS]**.  [24/203 vs. 37/318 in intervention and control groups, respectively]. |

References

1. Sibley LM, Sipe TA, Brown CM, Diallo MM, McNatt K, Habarta N: **Traditional birth attendant training for improving health behaviours and pregnancy outcomes**. *Cochrane Database Syst Rev* 2007(3):CD005460.

2. Jokhio AR, Winter HR: **An Intervention Involving TBAs and Perinatal and Maternal Mortality in Pakistan**. *N Engl J Med* 2005, **352**:2091-2099.

3. O'Rourke K: **The effect of a traditional birth attendant training program on obstetrical practices and perinatall mortality in rural Guatemala [dissertation]**. Amerherst: University of Massachusetts; 1994.
