# Supplementary material for: Delivering interventions to reduce the global burden of stillbirths: improving service supply and community demand
Source: BMC Pregnancy Childbirth. 2009 May 7;9(Suppl 1):S7. doi: 10.1186/1471-2393-9-S1-S7 (PMC2679413; doi:10.1186/1471-2393-9-S1-S7)
Supplement: Additional file 2 — Web Table 2. Component studies in Hodnett and Fredericks 2003 meta-analysis: Impact of support during pregnancy by health workers and midwives on stillbirth/neonatal mortality. Component studies in Hodnett and Fredericks 2003 meta-analysis reporting impact on stillbirths/perinatal mortality. [file 1471-2393-9-S1-S7-S2.doc]

**Web Table 2. Component studies in Hodnett and Fredericks 2003 [1] meta-analysis: Impact of support during pregnancy by health workers and midwives on stillbirth/neonatal mortality**

| **Source** | **Location and Type of Trial** | **Intervention** | **Stillbirths/Perinatal Outcomes** |
| --- | --- | --- | --- |
| 1. Blondel et al. 1990 [2] | France. Maternity units.  RCT. 158 pregnant women with moderate threatened preterm labour between 26-36 weeks' gestation, no IV betamimetics. | Compared the impact on pregnancy outcomes of intervention involving 1-2 home visits/week by midwives and access to domiciliary midwives via telephone, plus routine care vs. routine care only from obstetricians or midwives at outpatient clinics, no home visits, and hospitalisation if necessary (controls). | SBR/NMR: RR=2.00 (95% CI: 0.19-21.61) **[NS]**.  [2/79 vs. 1/79 in intervention and control groups, respectively]. |
| 2. Bryce et al. 1991 [3] | Australia (Perth).  RCT. N=1970 women with history of one or more preterm births, one or more low birthweight births, one or more perinatal deaths, three or more first trimester miscarriages, one or more second trimester miscarriages, or an antepartum hemorrhage in a previous pregnancy. | Compared the impact on pregnancy outcomes of intervention that included routine care plus home visits to provide sympathy, understanding, acceptance, and affection at approximately 4-6 week intervals (more frequently if the woman desired) and in-between telephone calls by midwives. The control group received routine antenatal care (not described). | SBR/NMR: RR=1.37 (95% CI: 0.80-2.36) **[NS]**.  [30/983 vs. 22/987 in the intervention and control groups, respectively]. |
| 3. Dawson et al. 1999 [4] | UK (Cardiff, South Wales).  RCT. N=60 pregnant women at varying stages of pregnancy, with a risk factor for low birth weight baby, e.g. hypertension, IUGR, isolated small antepartum bleeds, or previous perinatal loss, which would ordinarily have led to hospital admission but not to immediate intervention. | Assessed the effect on pregnancy outcomes of intervention of an average of 11 home visits by midwives plus a telephone domiciliary fetal monitoring system. The control group had conventional hospital care (not described). | SBR/NMR: RR=0.88 (95% CI: 0.06-13.65) **[NS]**.  [1/43 vs. 1/38 in intervention and control groups, respectively]. |
| 4. Heins et al. 1990 [5] | USA (South Carolina). State-funded antenatal clinics.  RCT. 1458 low-income pregnant women at varying gestations, free of known medical or pregnancy complications, score > 9 on a risk factors scale for low birth weight baby or had a low birth weight infant in the previous pregnancy. | Compared the impact on stillbirth/neonatal mortality of the intervention of weekly or biweekly antenatal care by a nurse-midwife, including education, counseling, assessment of the cervix, and screening. The control group had usual antenatal care (not described). | SBR/NMR: RR=0.30 (95% CI: 0.08-1.09) **[NS]**.  [3/728 vs. 10/730 in intervention and control groups, respectively]. |
| 5. Klerman et al. 2001[6] | USA (Alabama, Jefferson County).  RCT. N=656 African American women who sought prenatal care from the Department of Health. | Assessed the impact on pregnancy outcomes of the experimental group in which care was aimed at informing pregnant women of their risks and what behaviours might improve pregnancy outcome. Women were given prenatal vitamins, offered a structured smoking cessation/reduction program, and offered regular meetings with a social worker, to reduce stress and strengthen existing social support networks. Prenatal appointments were every two weeks, with minimum waiting times, on-site child care, evening hours, and transportation. In addition, each visit included a group educational session. The control group was given usual care by the county health department or the university's obstetrics dept. No specific educational or support programs. | SBR/NMR: RR=1.33 (95% CI: 0.43-4.13) **[NS]**.  [7/318 vs. 5/301 in the experimental and control groups, respectively]. |
| 6. Oakley et al. 1990 [7] | UK. Antenatal clinics of 4 hospitals.  RCT. N=509 women with a history of a low birth weight (< 2500 gm) baby, < 24 weeks gestation, singleton pregnancy, and fluent in English. | Compared the impact on pregnancy outcome of intervention involving usual antenatal care plus social support by the research midwife at her hospital. The social support intervention consisted of, at a minimum, 3 home visits - at 14, 20, and 28 weeks' gestation - plus 2 telephone contacts or brief home visits between these times. The midwife was also on-call to the mothers 24 hours/day. Semi-structured interview guides provided the basis for flexible and open-ended communication between midwives and mothers. The control group received usual antenatal care. | SBR/NMR: RR=1.66 (95% CI: 0.40-6.87) **[NS]**.  [5/255 vs. 3/254 in intervention and control groups, respectively]. |
| 7. Rothberg 1991a[8-11] | South Africa (Soweto). Obstetric clinics.  RCT. N=80 poor black pregnant women with hypertension and < 26 weeks' gestation booked for delivery at Baragwanath Maternity Hospital, Johannesburg. | Assessed the effect on stillbirth/neonatal mortality of intervention comprising of counseling by a social worker either at the time of a clinic visit, in a group session, or in a home visit (or hospital visit if the mother was hospitalised), on average approximately 4 times during the remainder of the pregnancy. The social worker provided psychosocial support and counseling, help with problems at home and at work, and encouragement to comply with clinic staff instructions/advice. The control group received routine care (not described) at the hypertension clinic and routine antenatal care. | SBR/NMR: RR=1.55 (95% CI: 0.72-3.32) **[NS]**.  [13/41 vs. 8/39 in intervention and control groups, respectively]. |
| 8. Rothberg et al. 1991b[8, 11, 12] | South Africa (Johannesburg).  RCT. N=104 Caucasian women with a singleton pregnancy | Assessed the effect on pregnancy outcome of intervention where a minimum of 20 minutes of individualised counseling from an assigned social worker at each antenatal visit or by telephone shortly thereafter was given. The control group received usual clinic care, in which personnel were largely unaware of mothers' personal problems. | SBR/NMR: RR=3.12 (95% CI: 0.13-74.76) **[NS]**.  [1/51 vs. 0/53 in intervention and control groups, respectively]. |
| 9. Spencer et al. (1989)[13-15] | England (South Manchester Health District).  RCT. N=1288 pregnant women < 20 weeks' gestation and at increased risk of giving birth to a low birth weight baby, booked for delivery in either of 2 maternity units. | Assessed the impact on pregnancy outcome on intervention of client-centred approach in which social support was provided by a family worker during pregnancy. The tasks of the worker varied according to the individual situation, and ranged from providing help in obtaining state benefits, with housing, shopping, and other domestic work and child care, to promoting appropriate use of health and social services and community facilities, and acting as a confidante. An average of 1-2 visits/week was provided. The control group received routine antenatal care (not described). | SBR/NMR: RR=1.69 (95% CI: 0.50-5.75) **[NS]**.  [7/655 vs. 4/633 in the intervention and control groups, respectively]. |
| 10. Spira et al. 1981 [16] | France.  RCT. N=996 women with pregnancy complications at risk for preterm delivery. | Compared the impact on stillbirth/neonatal mortality of domiciliary care by midwives (intervention) vs. hospitalization (controls). | SBR/NMR: RR=12.62 (95% CI: 0.71-223.40) **[NS]**.  [6/510 vs. 0/495 in intervention and control groups, respectively]. |
| 11. Villar et al. (1992) [17-21] | Argentina (Rosario), Brazil (Pelotas), Cuba (Havana) and Mexico City.  RCT. N=2235 pregnant women at risk for giving birth to a low birth weight baby, between 15-22 weeks' gestation. | Assessed the effect of the intervention aimed at increasing social support and reducing stress and anxiety in pregnancy. A minimum of 4 home visits by specially trained female social workers or obstetrical nurses. The aims of the visits were to strengthen the woman's social network, and to provide direct emotional support and health education. In addition, a special support office - for women to visit without prior appointments or to telephone - was available at each study hospital for all women in this group. The control group had standard antenatal care (not described) | SBR/NMR: RR=0.88 (95% CI: 0.57-1.37) [NS].  [37/1115 vs. 42/1120 in the intervention and control groups, respectively]. |

References

1. Hodnett ED, Fredericks S: **Support during pregnancy for women at increased risk of low birthweight babies**. *Cochrane Database of Systematic Reviews* 2003, **3**:CD000198.

2. Blondel B, Breart G, Llado J, Chartier M: **Evaluation of the home-visiting system for women with threatened preterm labor: results of a randomized controlled trial**. *European Journal of Obstetrics & Gynecology and Reproductive Biology* 1990, **34**:47-58.

3. Bryce RL, Stanley FJ, Garner JB: **Randomized controlled trial of antenatal social support to prevent preterm birth**. *Br J Obstet Gynaecol* 1991, **98**(10):1001-1008.

4. Dawson A, Cohen D, Candelier C, Jones G, Sanders J, Thompson A, Arnall C, Coles E: **Domiciliary midwifery support in high-risk pregnancy incorporating telephonic fetal heart rate monitoring: a health technology randomized assessment**. *J Telemed Telecare* 1999, **5**(4):220-230.

5. Heins HC, Jr., Nance NW, McCarthy BJ, Efird CM: **A randomized trial of nurse-midwifery prenatal care to reduce low birth weight**. *Obstet Gynecol* 1990, **75**(3 Pt 1):341-345.

6. Klerman LV, Ramey SL, Goldenberg RL, Marbury S, Hou J, Cliver SP: **A randomized trial of augmented prenatal care for multiple-risk, medicaid-eligible African American women**. *American Journal of Public Health* 2001, **91**:105-111.

7. Oakley A, Rajan L, Grant A: **Social support and pregnancy outcome**. *Br J Obstet Gynaecol* 1990, **97**(2):155-162.

8. Rothberg A: **Effects of stress and counselling on birthweight in two Johannesburg communities [PhD thesis]**. Johannesburg, South Africa: University of Witwatersrand; 1991.

9. Rothberg AD, Shuenyane E, Sefuba M: **Psychosocial support for mothers with pregnancy-related hypertension: effect on birthweight**. *Pediatric Reviews and Communications* 1991, **6**:13-20.

10. Rothberg AD SE, Lits B, Strebel PM **Effect of stress on birth weight in two Johannesburg populations**. *South African Medical Journal* 1991, **79**:35-38.

11. Rothberg AD LB, Shuenyane E: **Effects of counselling on birthweight in two Johannesburg communities**. In: *Proceedings of the 10th Conference on Priorities in Perinatal Care: 1991; South Africa*; 1991: 103-106.

12. Rothberg AD LB: **Psychosocial support for maternal stress during pregnancy: Effect on birth weight**. *American Journal of Obstetrics and Gynecology* 1991, **165**:403-407.

13. Spencer B, Thomas H, Morris J: **A randomized controlled trial of the provision of a social support service during pregnancy: the South Manchester Family Worker Project**. *Br J Obstet Gynaecol* 1989, **96**(3):281-288.

14. Spencer B: **The family workers project: evaluation of a randomized controlled trial of a pregnancy social support service**. In: *Proceedings of International Symposium on Advances in the Prevention of Low Birthweight: 1988 May 8-11; Cape Cod, Massachusetts, USA*; 1988 May 8-11: 109-121.

15. Spencer B, Morris J: **The family worker project: social support in pregnancy**. In: *Prevention of preterm birthVol 138.* Edited by Papiernik E, Breart G, Spira N. Paris: Colloque INSERM; 1986: 363-382.

16. Spira N, Audras F, Chapel A, Debuisson E, Jacquelin J, Kirchhoffer C, Lebrun C, Prudent C: **[Domiciliary care of pathological pregnancies by midwives. Comparative controlled study on 996 women (author's transl)]**. *J Gynecol Obstet Biol Reprod (Paris)* 1981, **10**(6):543-548.

17. Villar J, Farnot U, Barros F, Victora C, Langer A, Belizan JM: **A randomized trial of psychosocial support during high-risk pregnancies. The Latin American Network for Perinatal and Reproductive Research**. *N Engl J Med* 1992, **327**(18):1266-1271.

18. Belizan JM, Barros F, Langer A, Farnot U, Victora C, Villar J: **Impact of health education during pregnancy on behavior and utilization of health resources. Latin American Network for Perinatal and Reproductive Research**. *Am J Obstet Gynecol* 1995, **173**(3 Pt 1):894-899.

19. Langer A GC, Leis T, Reynoso S, Hernandez B: **Psychosocial support in pregnancy as a strategy to promote the newborn's health (translation)**. *Revista de Investigacion Clinica* 1993, **45**:317-328.

20. Langer A, Victora C, Victora M, Barros F, Farnot U, Belizan J, et al: **The Latin American trial of psychosocial support during pregnancy: a social intervention evaluated through an experimental design.** *Social Science and Medicine* 1993, **36**:495-507.

21. Victora CG, Langer A, Barros F, Belizan J, Farnot U, Villar J: **The Latin American Multicenter Trial on psychosocial support during pregnancy: methodology and baseline comparability. Latin American Network for Perinatal and Reproductive Research (LANPER)**. *Control Clin Trials* 1994, **15**(5):379-394.
