# Supplementary material for: Delivering interventions to reduce the global burden of stillbirths: improving service supply and community demand
Source: BMC Pregnancy Childbirth. 2009 May 7;9(Suppl 1):S7. doi: 10.1186/1471-2393-9-S1-S7 (PMC2679413; doi:10.1186/1471-2393-9-S1-S7)
Supplement: Additional file 3 — Web Table 3. Component studies in Villar et al. 2001 meta-analysis: Impact of different patterns of antenatal care on perinatal mortality. Component studies in Villar et al. 2001 meta-analysis reporting impact on stillbirths/perinatal mortality. [file 1471-2393-9-S1-S7-S3.doc]

**Web Table 3. Component studies in Villar et al. 2001 [1] meta-analysis: Impact of different patterns of antenatal care on perinatal mortality**

| **Source** | **Location and Type of Study** | **Intervention** | **Stillbirths / Perinatal Outcomes** |
| --- | --- | --- | --- |
| 1. Tucker et al. 1996 [2, 3] | Scotland. Multicentered (9 maternity hospitals).  RCT. N=1765 women. | Compared the impact on perinatal mortality of routine antenatal care by general practitioners and midwives according to a care plan and protocols for managing complications (intervention) vs. standard shared care between midwives and obstetricians (controls). | PMR: OR=0.76 (95% CI: 0.26-2.16) **[NS]**.  [6/834 vs. 8/840 in intervention and control groups, respectively]. |
| 2. Turnbull et al. 1996 [4-7] | UK. Hospital-based consultant clinics.  RCT. N=1299 women booking for care. | Assessed the effect on perinatal mortality of midwife managed care with provision of visit with specialist if needed (intervention) vs. shared care (controls). | PMR: OR=0.45 (95% CI: 0.15-1.35) **[NS]**.  [4/613 vs. 9/603 in intervention and control groups, respectively]. |

References

1. Villar J, Carroli G, Khan-Neelofur D, Piaggio G, Gulmezoglu M: **Patterns of routine antenatal care for low-risk pregnancy**. *Cochrane Database Syst Rev* 2001(4):CD000934.

2. Tucker JS, Hall MH, Howie PW, Reid ME, Barbour RS, Florey C du V, et al: **Should obstetricians see women with normal pregnancies? A multicentre randomised controlled trial of routine antenatal care by general practitioners and midwives compared with shared care led by obstetricians**. *BMJ* 1996, **312**:554-559.

3. Ratcliffe J, Ryan M, Tucker J: **The costs of alternative types of routine antenatal care for low-risk women: shared care vs care by general practitioners and community midwives**. *J Health Serv Res Policy* 1996, **1**(3):135-140.

4. Turnbull D, Holmes A, Shields N, Cheyne H, Twaddle S, Gilmour WH, McGinley M, Reid M, Johnstone I, Geer I *et al*: **Randomised, controlled trial of efficacy of midwife-managed care**. *Lancet* 1996, **348**(9022):213-218.

5. Shields N, Turnbull D, Reid M, Holmes A, McGinley M, Smith LN: **Satisfaction with midwife-managed care in different time periods: a randomised controlled trial of 1299 women**. *Midwifery* 1998, **14**(2):85-93.

6. Cheyne H, McGinley M, Turnbull D, Holmes A, Shields N, Greer I, et al: **Midwife managed care: results of a randomised controlled trial of 1299 women**. *Prenatal and Neonatal Medicine* 1996, **1**:129.

7. Turnbull D, Holmes A, Cheyne H, Shields N, McGinley M, McIlwaine G, et al: **Does midwife-led care work? The results of randomised controlled trial of 1299 women**. *27th British Congress of Obstetrics and Gynaecology* 1995:527.
