# Supplementary material for: Delivering interventions to reduce the global burden of stillbirths: improving service supply and community demand
Source: BMC Pregnancy Childbirth. 2009 May 7;9(Suppl 1):S7. doi: 10.1186/1471-2393-9-S1-S7 (PMC2679413; doi:10.1186/1471-2393-9-S1-S7)
Supplement: Additional file 4 — Web Table 4. Component studies in Hodnett 2000 meta-analysis: Impact of continuous care by caregivers during pregnancy and childbirth on stillbirth/neonatal mortality. Component studies in Hodnett 2000 meta-analysis reporting impact on stillbirths/perinatal mortality. [file 1471-2393-9-S1-S7-S4.doc]

**Web Table 4. Component studies in Hodnett 2000 [1] meta-analysis: Impact of continuous care by caregivers during pregnancy and childbirth on stillbirth/neonatal mortality**

| **Source** | **Location and Type of Study** | **Intervention** | **Stillbirths / Perinatal Outcomes** |
| --- | --- | --- | --- |
| 1. Flint et al. 1989 [2] | UK (London). Hospital-based.  RCT. N=1001 women who were booked for delivery at the hospital (N=503 intervention group, N=498 controls). | Compared the impact on pregnancy outcomes of intervention consisting of antenatal, intrapartum, and postnatal care by a team of four midwives, as well as consultation with an obstetrician at 36 and 41 weeks (if applicable) and as needed. The control women got routine care by a variety of caregivers (physicians and midwives) during pregnancy, childbirth, and the postnatal period. | SBR/NMR: OR=1.94 (95% CI: 0.62-6.06) **[NS]**.  [8/503 vs. 4/498 in intervention and control groups, respectively]. |
| 2. Rowley et al. 1995 [3] | Australia (New South Wales). Hospital based.  RCT. N=814 women booked for delivery at the hospital. (N=405 intervention group, N=409 controls). | Compared the impact on pregnancy outcomes of intervention group receiving antenatal care, one-to-one intrapartum care, and early postnatal care by a team of six experienced and newly graduated midwives. Low-risk women had 3 scheduled consultations with an obstetrician, and additional consultations as needed. High-risk women had consultations with an obstetrician at a frequency determined according to their needs. The control group received care from a variety of physicians and midwives working in the antenatal clinic, the delivery suite, and the postnatal ward. | SBR/NMR: OR=1.98 (95% CI: 0.53-7.36) **[NS]**.  [6/405 vs. 3/409 in intervention and control groups, respectively]. |

References

1. Hodnett ED: **Continuity of caregivers for care during pregnancy and childbirth**. *Cochrane Database Syst Rev* 2000(2):CD000062.

2. Flint C, Poulengeris P, Grant A: **The 'Know Your Midwife' scheme--a randomised trial of continuity of care by a team of midwives**. *Midwifery* 1989, **5**(1):11-16.

3. Rowley MJ, Hensley MJ, Brinsmead MW, Wlodarczyk JH: **Continuity of care by a midwife team versus routine care during pregnancy and birth: a randomised trial**. *Med J Aust* 1995, **163**(6):289-293.
