# Supplementary material for: Delivering interventions to reduce the global burden of stillbirths: improving service supply and community demand
Source: BMC Pregnancy Childbirth. 2009 May 7;9(Suppl 1):S7. doi: 10.1186/1471-2393-9-S1-S7 (PMC2679413; doi:10.1186/1471-2393-9-S1-S7)
Supplement: Additional file 5 — Web Table 5. Component studies in Olsen et al. 1997 meta-analysis: Impact of planned home versus hospital births on perinatal mortality. Component studies in Olsen et al. 1997 meta-analysis reporting impact on stillbirths/perinatal mortality. [file 1471-2393-9-S1-S7-S5.doc]

**Additional file 5 - Web Table 5. Component studies in Olsen et al. 1997 [1] meta-analysis: Impact of planned home versus hospital births on perinatal mortality**

| **Source** | **Location and Type of Study** | **Intervention** | **Stillbirths / Perinatal Outcomes** |
| --- | --- | --- | --- |
| Ackermann-Liebrich et al. 1994 [2] | Switzerland (Zurich).  Observational study. N=874 women (N=489 exposed group, N=385 unexposed).women intending hospital delivery. | Compared the impact on perinatal mortality among women intending home delivery (exposed) vs. hospital delivery (unexposed). | PMR: 1/489 vs. 1/385 in exposed and unexposed groups, respectively. (PMR Zurich 1990: 7.9/1000).  . |
| Berghs and Spanjaards 1988 [3, 4] |  | | |
| Durand 1992[5] | USA (Tennessee). Rural setting.  Cohort study. N=1707 women in the exposed group. | Compared the impact on perinatal mortality of midwife attended home births from the Farm midwifery service (exposed) to a sample of physician-attended hospital births derived from the 1980 US National Natality/National Fetal Mortality Survey (NNS/NFMS) (controls). | Fetal (miscarriage + SB) and neonatal death, labor-related complications, or low 5-minute Apgar scores: no significant differences between the two groups, regardless of whether the comparisons  were crude or adjusted for confounders. |
| Mehl 1977[6, 7] | USA.  Observational study. N=2,092 women (N=1046 home deliveries, N=1046 hospital deliveries). | Compared the impact on perinatal mortality of home (study group) versus hospital delivery (controls). | PMR: 2.9/1000 vs. 1.9/1000 in study vs. control groups, respectively **[NS]**. |
| Shearer 1985[8] | Country ? (Essex).  Observational study. N=387 (N=202 study group, N=185 controls). | Compared the impact on perinatal mortality of booking for a home confinement (study group) vs. hospital delivery under consultant care (controls). | PMR: none in either group. |
| Woodcock et al. 1994 [9] | Western Australia.  Retrospective cohort analysis. N=3,904 women 1981-87 with singleton pregnancies (N=976 study group, N=2928 controls). | Compared the impact on perinatal mortality of women planning a home birth (study group) vs. a planned hospital birth (controls). | PMR: OR=1.25 (95% CI: 0.44-3.55). |

# References

1. Olsen O: **Meta-analysis of the safety of home birth**. *Birth* 1997, **24**(1):4-13; discussion 14-16.

2. Ackermann-Liebrich U, Guenter-Witt K, Zuellig M, Kunz I, Voegel T: **Comparing home to hospital deliveries: recruitment, referrals and neonatal outcome**. *Soz Praventivmed* 1994, **39**(1):28.

3. Berghs G, Spanjaards E: **De Normale Zwangerschap: Bevallingen Beleid (The normal pregnancy: Delivery with thoughtfulness)**. Nijmegen, Netherlands; 1988.

4. Croons M: **Hjemmef0dsler i Holland (Home birth in Holland)**. *HjemmefGdsler og Livskunst (Home birth and the art of living)* 1995, **4**:44-53.

5. Duran AM: **The safety of home birth: the farm study**. *Am J Public Health* 1992, **82**(3):450-453.

6. Mehl L: **Research on alternatives in childbirth-what can it tell us about hospital practice?** . In: *2Ist Century Obstetrics Now! .* Edited by Stewart L SD, eds, vol. 1. Marble Hill, MO: NAPSAC; 1977: 171-207.

7. Mehl L: **The outcome of home delivery research in the United States**. In: *The Place of Birth.* Edited by Kitzinger S DJ, eds. Oxford: Oxford Medical Publications; 1978: 93-117.

8. Shearer JM: **Five year prospective survey of risk of booking for a home birth in Essex**. *Br Med J (Clin Res Ed)* 1985, **291**(6507):1478-1480.

9. Woodcock HC, Read AW, Bower C, Stanley FJ, Moore DJ: **A matched cohort study of planned home and hospital births in Western Australia 1981-1987**. *Midwifery* 1994, **10**(3):125-135.
