# Supplementary material for: Delivering interventions to reduce the global burden of stillbirths: improving service supply and community demand
Source: BMC Pregnancy Childbirth. 2009 May 7;9(Suppl 1):S7. doi: 10.1186/1471-2393-9-S1-S7 (PMC2679413; doi:10.1186/1471-2393-9-S1-S7)
Supplement: Additional file 6 — Web Table 6. Component studies in Hodnett et al. 2005 meta-analysis: Impact of home-like versus conventional settings for birth on perinatal mortality. Component studies in Hodnett et al. 2005 meta-analysis reporting impact on stillbirths/perinatal mortality. [file 1471-2393-9-S1-S7-S6.doc]

**Web Table 6. Component studies in Hodnett et al. 2005 [1] meta-analysis: Impact of home-like versus conventional settings for birth on perinatal mortality**

| **Source** | **Location and Type of Study** | **Intervention** | **Stillbirths / Perinatal Outcomes** |
| --- | --- | --- | --- |
| 1. Byrne2000 [2] | Australia (Adelaide). Queen Victoria Hospital.  RCT. N=201 nulliparous and multiparous women booked for delivery. | Compared the impact on perinatal mortality of women allocated to birth centre care with antenatal, intrapartum, and up to 12 hours of intrapartum care from midwives who were "committed to the normality of the birth process". Intrapartum care may have been by midwives who were not known to the women. The women were also encouraged to attend 2 classes about the birthing centre. The birthing centre consisted of 2 homelike rooms adjacent to the delivery suite, staffed by midwives (intervention). The control group received usual care antenatal care and their intrapartum care was in the conventional delivery suite; they were under the care of both a midwife and doctor. | PMR: RR=not estimable.  [0/100 vs. 0/101 in intervention and control groups, respectively]. |
| 2. Hundley 1994 [3-6] | Scotland (Aberdeen). Hospital-based.  RCT. N=2844 nulliparous and multiparous women low-risk at booking (N=1900 intervention group, N=944 controls). | Compared the impact on perinatal mortality of antenatal care and delivery performed in midwife-managed, home-like unit 20 yards from the hospital's delivery suite. The midwives' unit was staffed by hospital midwives who also worked in the delivery suite. Strict protocols were in place for booking, admission, and transfer of women. Labour was managed with minimal intervention and fetal monitoring by intermittent auscultation. The control group received care in the consultant-led delivery suite. | PMR: RR=1.24 (95% CI: 0.48-3.19) **[NS]**.  [15/1900 vs. 6/944 in intervention and control groups, respectively]. |
| 3. Klein 1984 [7] | Canada (Montreal).  Quasi-randomised trial. N=114 nulliparous and multiparous women (N=56 intervention group, N=58 controls). | Compared the impact on perinatal mortality of intrapartum care in a home-like birth room (intervention) vs. standard care in an adjacent labour ward in a tertiary care hospital (contols).  The same medical and nursing staff provided care in both settings. | PMR: RR=not estimable.  [0/56 vs. 0/58 in intervention and control groups, respectively]. |
| 4. Mac Vicar 1993 [8] | UK (Leicester).  RCT. N=3510 nulliparous and multiparous women booked for delivery at a hospital (N=2304 intervention group, N=1206 controls). | Assessed the effect on perinatal mortality of the intervention of antenatal care that included routine care by the general practitioner or community midwife except for 3 scheduled visits to the clinic staffed by hospital midwives, and intrapartum care in a 3-room, home-like unit adjacent to the delivery suite, staffed by 10 staff midwives who were not normally involved with the care of women in the delivery suite. The control group received routine antenatal care and care in the delivery suite. The majority had antenatal care shared between a consultant and general practitioner or community midwife; a small number had antenatal care from the general practitioner and community midwife. | PMR: RR=1.88 (95% CI: 0.70-5.06) [NS].  [18/2304 vs. 5/1206 in intervention and control groups, respectively]. |
| 5. Waldenstrom 1997, Gottvall K, et al. [9-16] | Sweden (Greater Stockholm). Tertiary care setting.  RCT. N=1860 nulliparous and multiparous women (N=928 intervention group, N=932 controls). | Compared the impact on perinatal mortality of intervention where antenatal, intrapartum, and postnatal care were in a home-like birth centre located one floor below the ordinary labour ward of the hospital, with 1:1 midwife-woman ratio during labour, and discharge within 24 hours of the birth. The controls received antenatal care at neighbourhood antenatal clinics, intrapartum care in the hospital labour delivery suite (usually each midwife caring for more than 1 woman), and postnatal care for 3-4 days in the hospital postnatal ward. | PMR: RR=4.02 (95% CI: 0.86-18.87) [NS].  [8/928 vs. 2/932 in intervention and control groups, respectively]. |

References

1. Hodnett ED, Downe S, Edwards N, Walsh D: **Home-like versus conventional institutional settings for birth**. *Cochrane Database Syst Rev* 2005(1):CD000012.

2. Byrne JP, Crowther CA, Moss JR: **A randomised controlled trial comparing birthing centre care with delivery suite care in Adelaide, Australia**. *Aust N Z J Obstet Gynaecol* 2000, **40**(3):268-274.

3. Hundley VA, Cruickshank FM, Lang GD, Glazener CM, Milne JM, Turner M, Blyth D, Mollison J, Donaldson C: **Midwife managed delivery unit: a randomised controlled comparison with consultant led care**. *BMJ* 1994, **309**(6966):1400-1404.

4. Hundley VA, Cruickshank FM, Milne JM, Glazener CM, Lang GD, Turner M, Blyth D, Mollison J: **Satisfaction and continuity of care: staff views of care in a midwife-managed delivery unit**. *Midwifery* 1995, **11**(4):163-173.

5. Hundley VA, Donaldson C, Lang GD, Cruickshank FM, Glazener CM, Milne JM, Mollison J: **Costs of intrapartum care in a midwife-managed delivery unit and a consultant-led labour ward**. *Midwifery* 1995, **11**(3):103-109.

6. Hundley VA, Milne JM, Glazener CM, Mollison J: **Satisfaction and the three C's: continuity, choice and control. Women's views from a randomised controlled trial of midwife-led care**. *Br J Obstet Gynaecol* 1997, **104**(11):1273-1280.

7. Klein M, Papageorgiou A, Westreich R, Spector-Dunsky L, Elkins V, Kramer MS, Gelfand MM: **Care in a birth room versus a conventional setting: a controlled trial**. *Can Med Assoc J* 1984, **131**(12):1461-1466.

8. MacVicar J, Dobbie G, Owen-Johnstone L, Jagger C, Hopkins M, Kennedy J: **Simulated home delivery in hospital: a randomised controlled trial**. *Br J Obstet Gynaecol* 1993, **100**(4):316-323.

9. Waldenstrom U, Nilsson CA: **A randomized controlled study of birth center care versus standard maternity care: effects on women's health**. *Birth* 1997, **24**(1):17-26.

10. Gottvall K, Waldenstrom U: **Does birth center care during a woman's first pregnancy have any impact on her future reproduction?** *Birth* 2002, **29**(3):177-181.

11. Waldenstrom U: **Effects of birth centre care on fathers' satisfaction with care, experience of the birth, and adaptation to fatherhood**. *Journal of Reproductive and Infant Psychology;* 1999, **17**:357-368.

12. Waldenstrom U, Nilsson CA: **No effect of birth centre care on either duration or experience of breast feeding, but more complications: findings from a randomised controlled trial**. *Midwifery* 1994, **10**(1):8-17.

13. Waldenstrom U, Nilsson CA: **Experience of childbirth in birth center care. A randomized controlled study**. *Acta Obstet Gynecol Scand* 1994, **73**(7):547-554.

14. Waldenstrom U, Nilsson CA: **Women's satisfaction with birth center care: a randomized, controlled study**. *Birth* 1993, **20**(1):3-13.

15. Waldenstrom U, Nilsson CA, Winbladh B: **The Stockholm birth centre trial: maternal and infant outcome**. *Br J Obstet Gynaecol* 1997, **104**(4):410-418.

16. Wilhelmson B: **[First results from a randomized study: ABC for alternative childbirth]**. *Lakartidningen* 1993, **90**(3):180-182.
