# Supplementary material for: Delivering interventions to reduce the global burden of stillbirths: improving service supply and community demand
Source: BMC Pregnancy Childbirth. 2009 May 7;9(Suppl 1):S7. doi: 10.1186/1471-2393-9-S1-S7 (PMC2679413; doi:10.1186/1471-2393-9-S1-S7)
Supplement: Additional file 7 — Web Table 7. Component studies in Mancey-Jones and Brugha 1997: Impact of perinatal audit on perinatal mortality. Component studies in Mancey-Jones and Brugha 1997 meta-analysis reporting impact on stillbirths/perinatal mortality. [file 1471-2393-9-S1-S7-S7.doc]

**Web Table 7. Component studies in Mancey-Jones and Brugha 1997 [1]: Impact of perinatal audit on perinatal mortality**

| **Source** | **Location and Type of Study** | **Intervention** | **Stillbirth/Perinatal Outcomes** |
| --- | --- | --- | --- |
| Bugalho and Bergstrom 1993 [2] | Mozambique. Maputo Central Hospital.  An audit. N=134,408 births registered during the 10-year period 1982-1991. | To study the impact on perinatal mortality over the years of a routine of daily and weekly perinatal audit that was initiated in 1982. | SBR: 44% vs. 42% in 1983 and 1986, respectively **[NS]**.  PMR: ~20% reduction [68.5% vs. 84.7% in 1990 vs, 1983, respectively.] 11% reduction between 1983 and 1984.  Intrapartum SBR: 3.9% vs. 10% in 1990 vs. 1983., respectively (P<0.0005). |
| de Caunes et al. 1990 [3] | West Indies (Guadeloupe).  An audit. N=354 perinatal deaths from November 1983 through December 1985. | To assess the effect of perinatal mortality audit, along with other interventions like educational broadcasts and media announcements to increase public awareness about perinatal health issues. | PMR: 26/1000.  PMR: fell by 25% during audit. |
| De Muylder 1989 [4] | Zimbabwe. District hospital and clinics.  Routine, internal audit. N = 319 perinatal deaths over a 25-month review period. | Formal audit of perinatal deaths. | PMR: 30.6/1000 during 1984-86.  Major risk of perinatal death and avoidable factors detected in 75.6% of cases |
| Pattinson et al. 1995 a [5] | South Africa. Black urban population.  Descriptive study. N = 202 perinatal deaths of infants weighing > 1000 g. | To assess the ‘ICA solution’ of audit (identifying, classifying and grading avoidable factors) to improve perinatal care. | PMR: 26/1000 deliveries. |
| Pattinson et al. 1995 b [6] | South Africa (Pretoria).  Internal audit. | To assess the impact of the Perinatal Problem Identification Programme (PPIP) based on the ICA Solution system of audit. | PMR: 26.6 vs. 16.6/1000 in 1991-92 and 1994 respectively (a 38% reduction). |
| Ward et al. 1995 [7] | South Africa (Port Elizabeth). Central referral hospital and 2 district hospitals.  Retrospective audit. N=22,585 deliveries (N=1,060 perinatal deaths) between 1991-1992 of gestational age > 28 weeks, or birth weight ≥ 1000 g (if gestational age unknown). | To assess the impact of the perinatal audit over the two-year period utilizing the ICA (Identification, Cause, Avoidable Factor) solution system. | PMR: OR=0.76 (95% CI: 0.67-0.86); P<0.05.  [52.8/1000 vs. 40.6/1000 in 1991 and 1992 respectively (23% reduction)]. |
| Wilkinson 1991 [8] | South Africa (Lebowa). District hospital and clinics.  Routine, internal audit. All (N = 90) perinatal deaths over a 7-month period. | To study the impact of a perinatal mortality intervention study in 1989 which involved introducing a variety of strategies to eliminate avoidable deaths. | PMR: 41/1000 vs. 60/1000 in 1989 and 1988 respectively (P<0.05). |

References

1. Mancey-Jones M, Brugha RF: **Using perinatal audit to promote change: a review**. *Health Policy Plan* 1997, **12**(3):183-192.

2. Bugalho A, Bergstrom S: **Value of perinatal audit in obstetric care in the developing world: a ten-year experience of the Maputo model**. *Gynecol Obstet Invest* 1993, **36**(4):239-243.

3. de Caunes F, Alexander GR, Berchel C, Guengant JP, Papiernik E: **The Guadeloupean perinatal mortality audit: process, results, and implications**. *Am J Prev Med* 1990, **6**(6):339-345.

4. De Muylder X: **Perinatal mortality audit in a Zimbabwean district**. *Paediatr Perinat Epidemiol* 1989, **3**(3):284-293.

5. Pattinson RC, Makin JD, Shaw A, Delport SD: **The value of incorporating avoidable factors into perinatal audits**. *S Afr Med J* 1995, **85**(3):145-147.

6. Pattinson RC, de Jonge E, Pistorius LR, Howarth GR, de Wet H, Bremer P, Hay IT, Delport S: **Practical application of data obtained from a Perinatal Problem Identification Programme**. *S Afr Med J* 1995, **85**(3):131-132.

7. Ward HR, Howarth GR, Jennings OJ, Pattinson RC: **Audit incorporating avoidability and appropriate intervention can significantly decrease perinatal mortality**. *S Afr Med J* 1995, **85**(3):147-150.

8. Wilkinson D: **Perinatal mortality--an intervention study**. *S Afr Med J* 1991, **79**(9):552-553.
